# Exploiting mitochondrial dysfunction to overcome BRAF inhibitor resistance in advanced melanoma: the role of disulfiram as a copper ionophore

**DOI:** 10.1038/s41419-025-07766-y

**Published:** 2025-07-01

**Authors:** Bolun Zhao, Fazhan Ban, Yuehua Li, Qiong Shi, Sen Guo, Xiuli Yi, Huina Wang, Tianwen Gao, Chunying Li, Guannan Zhu

**Affiliations:** 1 Department of Dermatology, Xijing Hospital, Fourth Military Medical University, Xi’an, China; 2 Dermatology Hospital, Southern Medical University, Guangzhou, China

**Keywords:** Cancer therapeutic resistance, Energy metabolism

## Abstract

Resistance to targeted therapies poses a significant challenge in advanced melanoma with BRAF mutations. Even with a BRAF + MEK inhibitor combination, about 70% of patients experience disease progression within two years, highlighting the need for novel strategies beyond MAPK signaling inhibition. This study investigates whether mitochondrial dysfunction induced by the copper ionophore disulfiram (DSF) can effectively counteract resistance to BRAF inhibitors. We established two BRAF inhibitor (BRAFi)-resistant melanoma cell lines using BRAF mutant 451Lu and UACC62. In vivo experiments were conducted using subcutaneous implantation in nude mice. Cell viability and colony formation assays assessed treatment efficacy, while mitochondrial morphology was evaluated via transmission electron microscopy. Mitochondrial respiration was measured using a Seahorse metabolic analyzer, and oxidative stress was assessed through flow cytometry and confocal microscopy. RNA sequencing identified downstream factors regulated by intracellular copper levels, and the CRISPR-Cas9 system was used to knock out candidate genes in BRAFi-resistant cells for mechanistic validation. We provided evidence that DSF induced cell death in BRAFi-resistant melanoma in a copper-dependent manner, severely impairing mitochondrial structure and function through increased oxidative stress. RNA-seq and immunoblotting revealed that thioredoxin-interacting protein (TXNIP) expression significantly increased in response to DSF. TXNIP knockout reduced DSF-induced cytotoxicity by mitigating oxidative stress. These findings were supported by in vivo experiments. Furthermore, we demonstrated that the oxidative damage mediated by TXNIP involved its interaction with thioredoxin 2 (TRX2). In conclusion, targeting mitochondrial function with disulfiram effectively inhibits BRAFi-resistant melanoma cells, independent of MAPK signaling blockage. These results point to the potential of combining disulfiram with BRAF inhibitors as a promising strategy to overcome BRAFi resistance.

## Introduction

Among the known pathogenic genes of melanoma, BRAF is the most frequent mutant driver gene, endowing the tumor cells with a more aggressive phenotype by constitutively activating the MAPK pathway [1, 2]. Therefore, targeted therapeutic approaches with BRAFi (e.g., vemurafenib and dabrafenib) brought significant clinical benefits to cases harboring the BRAF V600E/K/R/D monomers (class I BRAF mutants) [3, 4]. However, soon after the application of BRAF inhibitors, drug resistance was observed in more than half of the patient cohort, even in those with initially high treatment response [5–7]. Given the predominant contribution of MAPK signaling reactivation in the BRAFi resistance [8, 9], the dual combination of MEK inhibitor with BRAF inhibitor was developed to overcome the situation. Unfortunately, although this approach did delay the development of resistance, approximately 70% of patients still suffered from disease progression in 2 years [7, 10]. Therefore, the development of novel strategies beyond the MAPK pathway inhibition for overcoming the BRAFi resistance is still needed.

Recent studies have demonstrated a new mechanism underlying BRAFi resistance involving enhanced mitochondrial network fusion and oxidative phosphorylation capacity [11, 12]. Preclinical attempts using mitochondrial inhibitors [13, 14] and mitochondria uncouplers [15] showed inhibition effects on melanoma cells resistant to MAPK inhibitors, suggesting the induction of mitochondrial dysfunction might be a promising strategy to overcome BRAFi resistance.

Among a variety of players in mitochondrial function and metabolism, we noticed the role of copper ion due to its versatility in mitochondrial bioenergetics, dynamics, and metabolic reprogramming [16, 17]. Moreover, with the emerging information regarding copper homeostasis in cancer biology, copper ionophores, including disulfiram, are believed to be potent anticancer agents for gastric cancer [18], thyroid cancer [19], liver cancer [20], and breast cancer [21].

In the current study, we examined the hypothesis that DSF reverses the resistance of melanoma cells to BRAFi by inducing mitochondrial dysfunction. Furthermore, we proved that the key mechanism of DSF-induced mitochondrial dysfunction is attributed to the oxidative damage dependent on intracellular copper overload. In addition, we revealed for the first time that TXNIP-TRX2 interaction is largely responsible for the mitochondrial oxidative damage by DSF. These results suggest that the combination of DSF with BRAFi may be a promising strategy to overcome BRAFi resistance.

## Results

### DSF inhibits BRAFi-resistant melanoma growth in a copper-dependent way

**Fig. 1:**
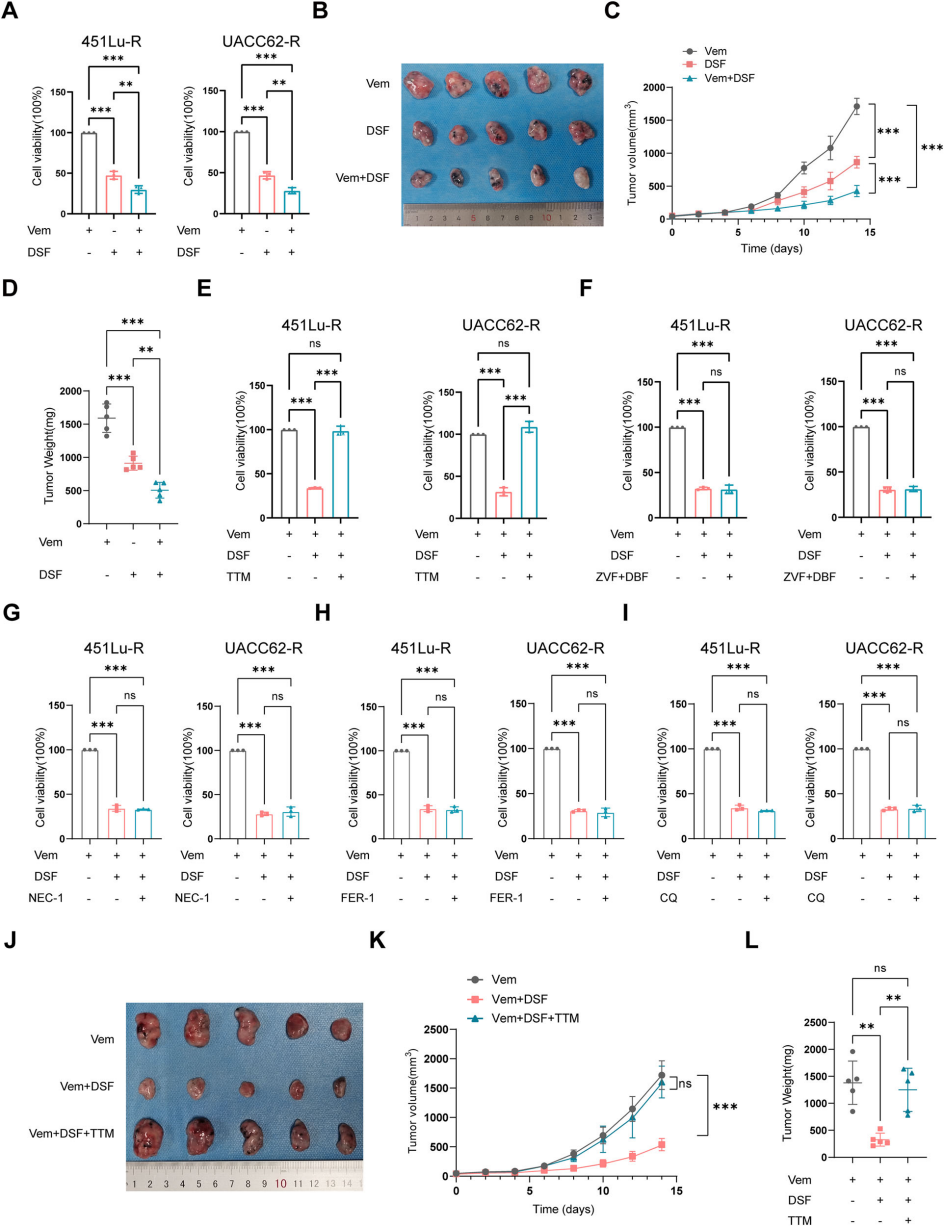
DSF inhibits BRAFi-resistant melanoma growth in a copper-dependent way. **A** CCK-8 assay of cell viability of two vemurafenib-resistant cell lines treated with vemurafenib (Vem) alone, disulfiram (DSF) alone, or Vem in combination with DSF for 48 h. **B**–**D** Images of tumors from mice that received Vem alone, DSF alone, or Vem in combination with DSF (**B**). Tumor volumes and weights in each group were measured and displayed in (**C**) and (**D**). **E** Viability of vemurafenib-resistant cells with and without pretreatment of 20 μM TTM exposed to Vem or Vem+DSF. **F** Viability of vemurafenib-resistant cells pretreated with 30 μM Z-VAD-FMK (ZVF) and 50 μM D-Boc-FMK(DBF), followed by treatment with Vem or DSF+Vem. **G** Viability of vemurafenib-resistant cells pretreated with 20 μM Necrostatin-1 (NEC-1), followed by treatment with Vem or DSF+Vem. **H** Viability of vemurafenib-resistant cells pretreated with 10 μM ferrostatin-1 (FER-1), followed by treatment with Vem or DSF+Vem. **I** Viability of vemurafenib-resistant cells pretreated with 5 μM chloroquine (CQ), followed by treatment with Vem or DSF+Vem. **J**–**L** Images of tumors from mice that received the indicated treatment (**J**). Tumor volumes and weights in each group were measured and displayed in (**K**) and (**L**). The symbol of one dot indicates one tumor sample, and the error bars are the mean ± SD. The differences were analyzed using one-way ANOVA. *P < 0.05, **P < 0.01, and ***P < 0.001. ns non-significant, ZVF Z-VAD-FMK, BDF Boc-D-FMK, NEC-1 Necrostatin-1, FER-1 Ferrostatin-1, CQ chloroquine.

Two melanoma cell lines carrying the BRAF^V600E^ mutations, 451Lu and UACC62, were used to generate vemurafenib-resistant (VR) cells. The resistance was determined by increased IC50 values (Supplementary Fig. 1A). We first treated these resistant cells with vemurafenib 5 μM, or DSF 1.5 μM, or their combination and observed that the combination of Vem+DSF exhibited a remarkably additive effect relative to either Vem or DSF alone (Fig. 1A). Likewise, a similar enhanced inhibitory effect was validated in vivo (Fig. 1B–D). Importantly, the nude mouse xenograft model further demonstrated that with prolonged treatment duration, the DSF+Vem combination showed an increasingly significant inhibitory advantage compared to DSF monotherapy. Therefore, in subsequent investigations, we prioritized elucidating the therapeutic effects and underlying mechanisms of the combination of Vem+DSF.

To define the mechanism of cell death caused by DSF+Vem, we co-treated BRAFi-resistant cells with inhibitors of known cell death pathways, accompanied by DSF. We found tetrathiomolybdate (TTM), a copper chelator (Fig. 1E), remarkably abrogated the cell death induced by DSF+Vem, whereas apoptosis inhibitors (Z-VAD-FMK and Boc-D-FMK) (Fig. 1F), necroptosis inhibitor (NEC-1) (Fig. 1G), ferroptosis inhibitor (FER-1) (Fig. 1H), and autophagy inhibitor (chloroquine) (Fig. 1I) all failed to reverse the cytotoxicity, suggesting the potency of cell death caused by DSF+Vem is largely mediated by increasing the intracellular copper accumulation. Given that the activation of MEK is responsible for a majority of BRAFi resistance, we asked whether the inhibition effect of DSF is associated with the suppression of the MEK pathway. WB revealed that, unlike the combination of MEK inhibitor, trametinib, the combination of DSF did not affect the ERK phosphorylation of BRAFi-resistant cells (Supplementary Fig. 1B). Immunoblotting uncovered that the cleaved caspase 3 did not respond to DSF exposure, indicating the cell death induced by DSF is apoptosis-independent (Supplementary Fig. 1C). Moreover, in line with the in vitro findings, TTM reversed the tumor inhibitory effect of DSF combination in vivo (Fig. 1J–L).

### DSF reverses the resistance to BRAFi by inducing mitochondrial dysfunction

**Fig. 2:**
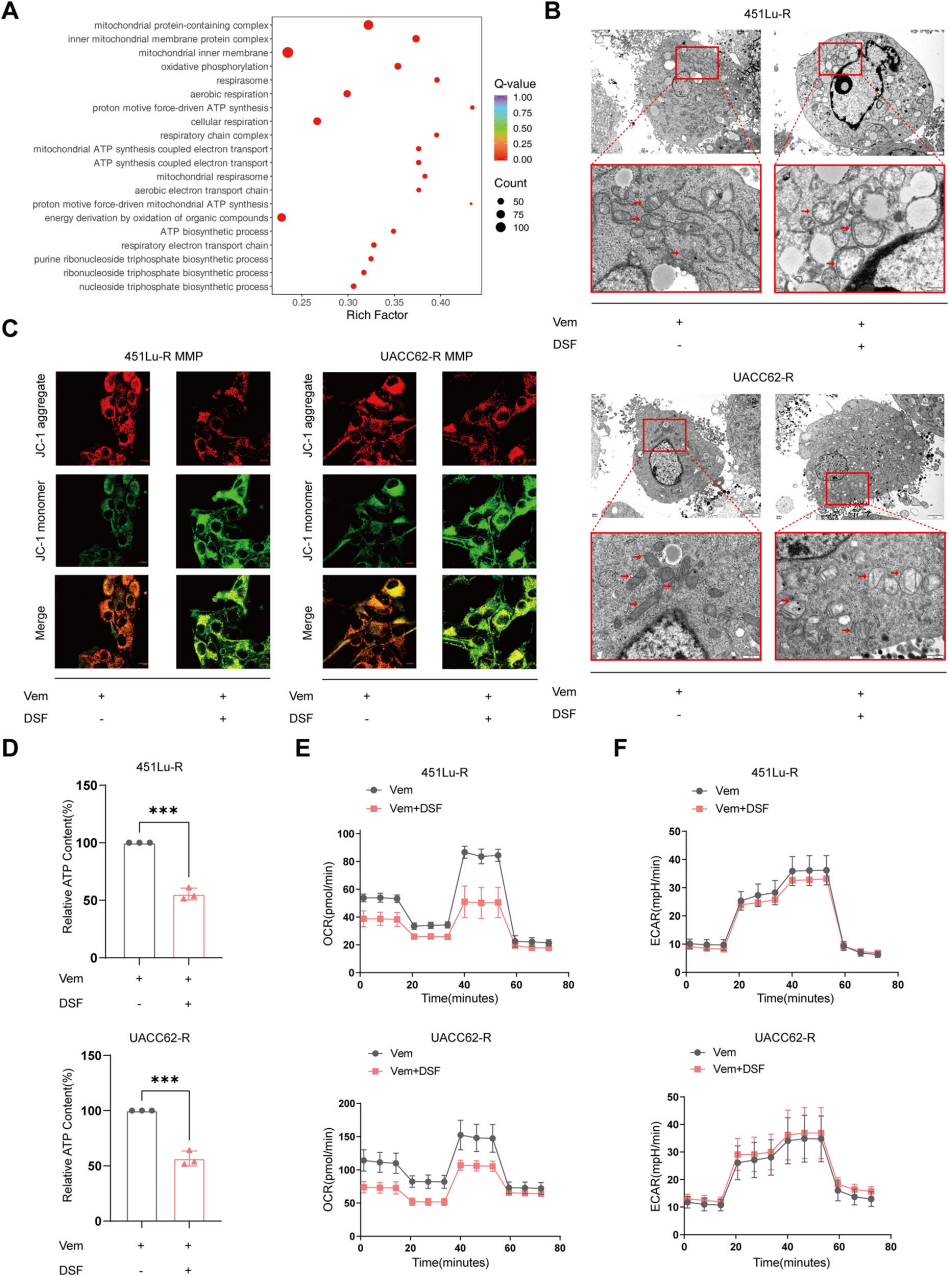
DSF reverses the resistance to BRAFi by inducing mitochondrial dysfunction. **A** RNA sequencing (RNA-seq) analysis of gene enrichment pathways comparing cells treated with Vem+DSF to cells treated with Vem. **B** Transmission electron microscopy (TEM) of mitochondrial morphology in cells treated with Vem or Vem+DSF for 48 h. **C** Immunofluorescence staining of mitochondrial membrane potential (MMP) in vemurafenib-resistant cells treated with Vem or Vem+DSF. **D** Detection of ATP levels in vemurafenib-resistant cells treated with Vem or Vem+DSF. **E**, **F** Seahorse assay to analyze mitochondrial oxidative phosphorylation (**E**) and glycolysis in vemurafenib-resistant cells Vem or Vem+DSF (**F**). Data are represented as mean ± SD of triplicate. *P-*value was calculated by two-­tailed Student’s *t*-­test. *P < 0.05, **P < 0.01, and ***P < 0.001. ns non-significant.

To further detect the mechanisms of Vem+DSF-induced cell death, we conducted RNA sequencing to compare the expression transcripts between BRAFi-resistant cells treated with Vem and Vem+DSF. The Gene Ontology (GO) analysis showed that differentially expressed genes were enriched in gene sets related to mitochondrial structure and function (Fig. 2A). It has been demonstrated that copper is not only essential for mitochondrial cuproenzymes assembling but also participates in mitochondrial function and dynamics [22–24]. We speculated that DSF may cause copper-dependent death of resistant cells by inducing mitochondrial dysfunction. Transmission electron microscopy (TEM) showed swollen mitochondria with vacuoles and/or abnormal cristae in BRAFi-resistant cells treated with Vem+DSF (Fig. 2B). Moreover, flow cytometry and laser confocal microscopy using JC-1 dye revealed a significant impairment of mitochondrial membrane potential (MMP) in Vem+DSF treated cells (Fig. 2C and Supplementary Fig. 2A). The ATP assay showed a reduction of ATP generation in cells treated with Vem+DSF (Fig. 2D). Furthermore, the seahorse assay revealed the mitochondrial oxidative respiration of VR cells was markedly suppressed by the combination of DSF with Vem, while the extracellular acidification rate (ECAR) was not affected (Fig. 2E, F and Supplementary Fig. 2B).

### Mitochondrial dysfunction caused by DSF+Vem depends on exacerbated oxidative stress

**Fig. 3:**
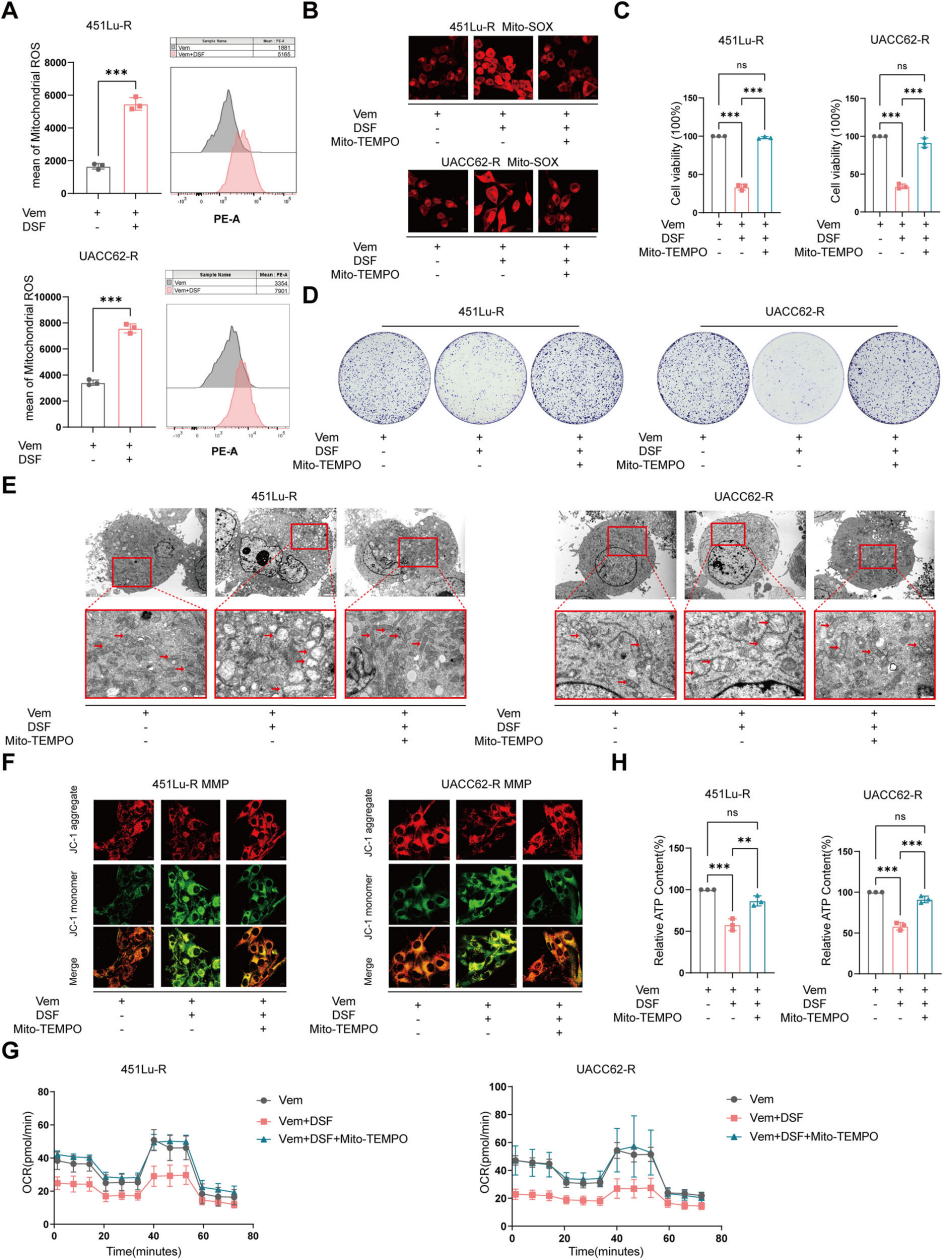
Mitochondrial dysfunction caused by DSF depends on exacerbated oxidative stress. **A** Flow cytometry staining was used to analyze mitochondrial reactive oxygen species (mt-ROS) levels. **B** Immunofluorescence staining was conducted to assess the mt-ROS level. **C** CCK-8 assay of cell viability of -resistant cells treated with Vem or DSF+Vem with or without Mito-TEMPO pretreatment. **D** Colony forming assay was used to describe the growth of cells pretreated with or without Mito-TEMPO, followed by Vem or DSF+Vem exposure. **E** TEM analyzed the mitochondrial morphology changes in vemurafenib-resistant cells pretreated with or without Mito-TEMPO, followed by Vem or DSF+Vem exposure. **F** Immunofluorescence staining of MMP levels. **G** Seahorse assay was used to assess the mitochondrial oxidative phosphorylation. **H** ATP levels of vemurafenib-resistant cells pretreated with or without Mito-TEMPO, followed by Vem or DSF+Vem exposure. Data represent the mean ± SD of triplicate. The differences were analyzed using one-way ANOVA. *P < 0.05, **P < 0.01, and ***P < 0.001. ns non-significant.

Recent studies linked cellular copper levels to mitochondrial dysfunction via exacerbated reactive oxygen species (ROS) production [25, 26]. To determine the contribution of oxidative stress to the cytotoxicity of BRAFi-resistant cells by DSF, we performed flow cytometry and found an excessive accumulation of mitochondrial ROS (mt-ROS) in drug-resistant cells exposed to Vem+DSF (Fig. 3A). Furthermore, we used Mito-TEMPO, a mitochondria-targeted antioxidant capable of specifically scavenging reactive oxygen species within the mitochondria, to clear the mt-ROS (Fig. 3B and Supplementary Fig. 3A). CCK-8 and clone formation assays demonstrated that Mito-TEMPO largely rescued the growth inhibition by DSF+Vem (Fig. 3C, D). Consistently, TEM photography, MMP assessment, the Seahorse assay, and ATP content assay (Fig. 3E–H, and Supplementary Fig. 3B, C), revealed that the mitochondrial dysfunction induced by DSF was restored by the pretreatment of Mito-TEMPO. Taken together, the above findings illustrate that DSF led to mitochondrial dysfunction by inducing oxidative stress damage.

### Depletion of copper rescues the DSF+Vem combination-induced mitochondrial dysfunction by reversing the oxidative damage

**Fig. 4:**
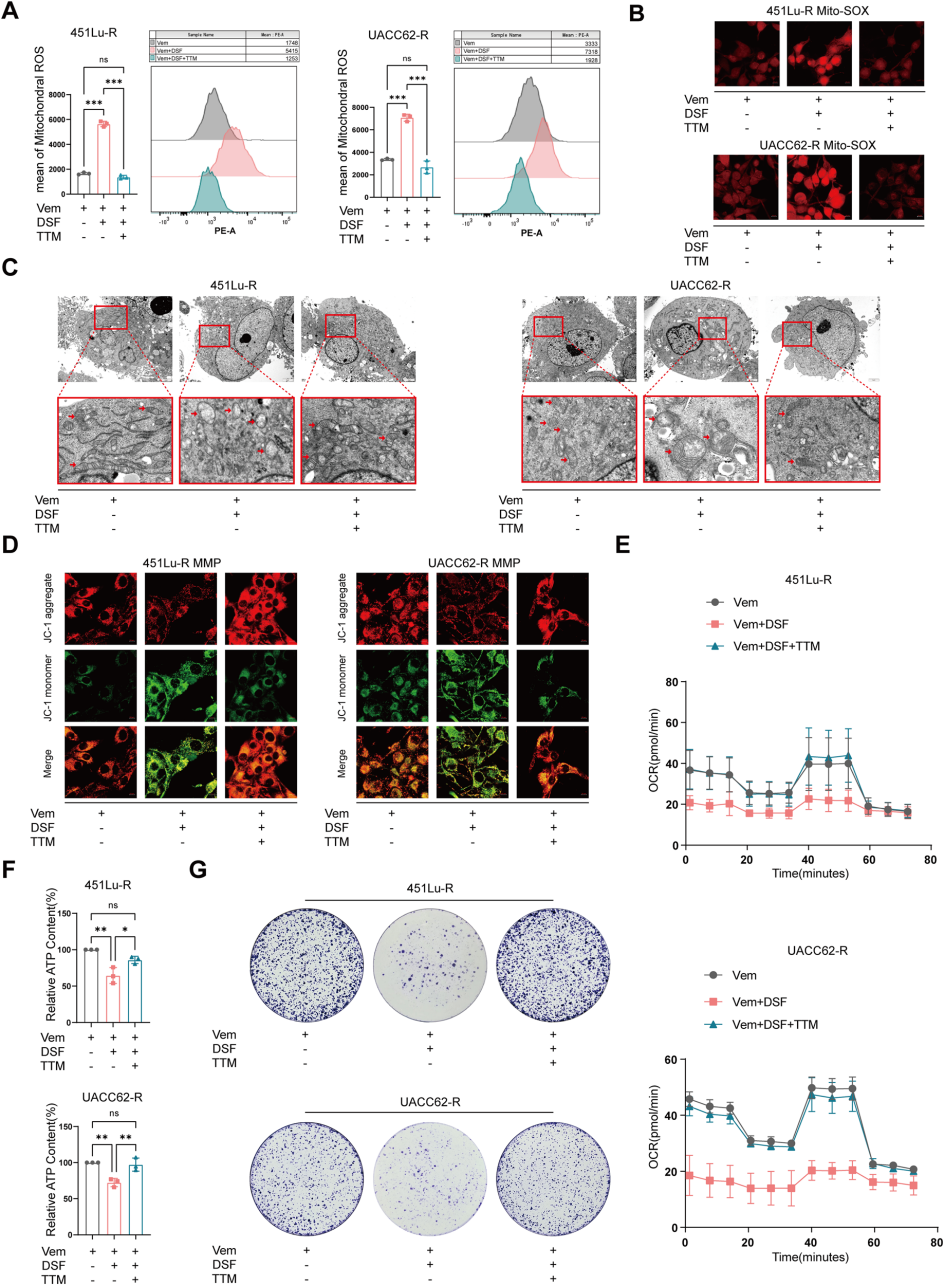
Depletion of copper rescues the DSF-induced mitochondrial dysfunction by reversing the oxidative damage. **A** Flow cytometry analysis of mt-ROS levels of vemurafenib-resistant cells pretreated with or without 20 μM TTM, followed by Vem or DSF+Vem exposure. **B** Immunofluorescence staining of mt-ROS levels. **C** TEM photography of the mitochondrial morphology of vemurafenib-resistant cells pretreated with or without 20 μM TTM, followed by Vem or DSF+Vem exposure. **D** Immunofluorescence staining of MMP levels. **E** Seahorse assay of vemurafenib-resistant cells pretreated with or without 20 μM TTM, followed by Vem or DSF+Vem exposure. **F** ATP level evaluation of cells pretreated with or without 20 μM TTM, followed by Vem or DSF+Vem exposure. **G** Colony formation assay vemurafenib-resistant cells were exposed to the indicated treatments. Data represent the mean ± SD of triplicate. The differences were analyzed using one-way ANOVA. *P < 0.05, **P < 0.01, and ***P < 0.001. ns non-significant.

To further confirm the role of copper in the mitochondrial oxidative damage by DSF, we performed the mitochondrial structure and function assessments following the pretreatment of TTM to deprive copper in the culture system. Firstly, flow cytometry (Fig. 4A) and confocal microscopy (Fig. 4B) showed that TTM significantly reduced the elevated mitochondrial oxidative stress of VR cells induced by DSF. Secondly, TEM photography revealed that the pretreatment of TTM maintained the mitochondria in a normal structure, characterized by uniform electron density of mitochondrial cristae and clearly defined straight cristae when exposed to Vem+DSF (Fig. 4C). Thirdly, cells pretreated with TTM showed significant resistance to the DSF-induced impairments of MMP (Fig. 4D and Supplementary Fig. 4A) and mitochondrial oxidative respiration (Fig. 4E, F, and Supplementary Fig. 4B). Additionally, in line with the cell viability assay (Fig. 1E), colony formation also indicated the rescue effect of TTM on suppressed VR cells by DSF+Vem (Fig. 4G). Given the role of cupper ionophore in inducing cuproptosis [17], we systematically examined the expression levels of cuproptosis-related molecules. WB analysis revealed that ATP7A/B expression was significantly upregulated, while SLC31A1, FDX1, LIAS, and DLAT expression were markedly downregulated in VR cells following Vem+DSF treatment (Supplementary Fig. 4C). Building upon the pivotal role of FDX1 in cuproptosis established by Professor Tsvetkov and colleagues [17, 27], we modulated FDX1 expression in drug-resistant cells. Notably, FDX1 knockdown failed to attenuate the synergistic inhibitory effect of DSF+Vem, while FDX1 overexpression did not enhance this combinatorial efficacy (Supplementary Fig. 4D–G), suggesting the existence of FDX1-independent pathways mediating DSF+Vem’s anti-tumor activity.

### TXNIP is responsible for the DSF+Vem combination-induced mitochondrial dysfunction

**Fig. 5:**
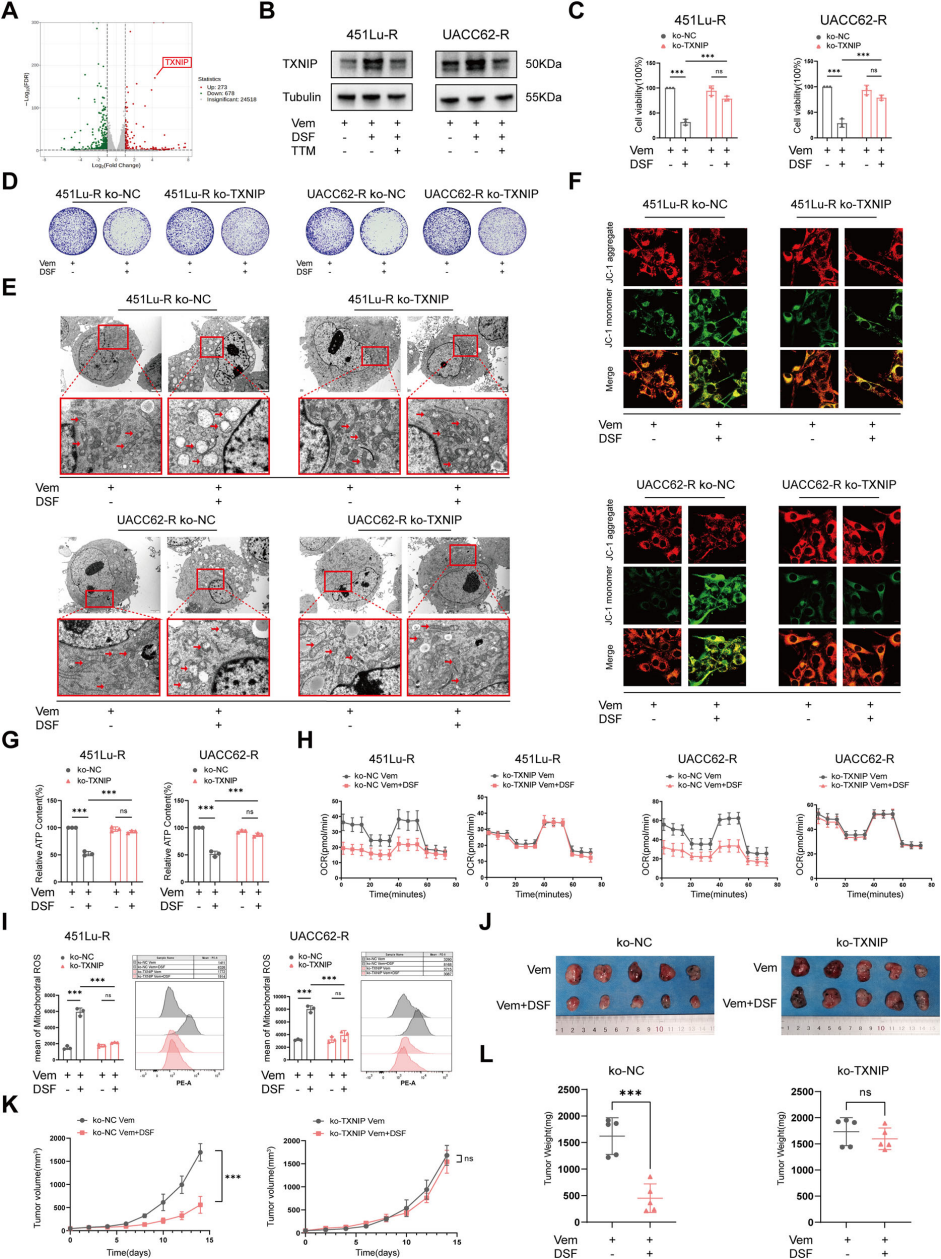
TXNIP is responsible for DSF-induced mitochondrial dysfunction. **A** Significantly changed genes by RNA-seq comparing vemurafenib-resistant cells treated with Vem+DSF to cells treated with Vem. **B** WB of TXNIP expression in vemurafenib-resistant cells exposed to Vem or Vem+DSF. **C** CCK-8 assay of vemurafenib-resistant cells with TXNIP knockout exposed to Vem+DSF or Vem. **D** Colony formation of vemurafenib-resistant cells with TXNIP knockout exposed to Vem+DSF or Vem. **E** TEM observation of vemurafenib-resistant cells with TXNIP knockout exposed to Vem+DSF or Vem. **F** Immunofluorescence staining of MMP levels in vemurafenib-resistant cells with TXNIP knockout exposed to Vem+DSF or Vem. **G** ATP content in vemurafenib-resistant cells with TXNIP knockout exposed to Vem+DSF or Vem. **H** Seahorse assay of vemurafenib-resistant cells with TXNIP knockout exposed to Vem+DSF or Vem. **I** Flow cytometry assay of mt-ROS levels in vemurafenib-resistant cells with TXNIP knockout exposed to Vem+DSF or Vem. **J**–**L** Tumors removed from nude mice bearing 451Lu-R ko-NC cells or 451Lu-R ko-TXNIP after 14 days of treatment with Vem+DSF or Vem (**J**). Tumor volumes and weights in each group were calculated and displayed in (**K**) and (**L**). Symbols of one dot indicate one mouse, and the error bars are mean ± SD. The differences were analyzed using Student’s *t*-test and one-way ANOVA. *P < 0.05, **P < 0.01, and ***P < 0.001. ns non-significant, ko-NC knockout negative control, ko-TXNIP knockout of TXNIP.

Next, we used the RNA sequencing data to identify the potential key player mediating the DSF+Vem combination-induced oxidative damage. Among the top significantly changed genes in response to the combination of DSF (Fig. 5A), we picked thioredoxin-interacting protein (TXNIP) as a candidate gene since its expression is closely associated with intracellular ROS level [26, 28]. WB and immunofluorescence staining were employed to confirm the expression of TXNIP. As shown in Fig. 5B and Supplementary Fig. 5A, the expression of TXNIP was significantly upregulated in cells treated with Vem+DSF, yet the change disappeared in cells pretreated with TTM, suggesting a positive correlation of copper level with TXNIP expression. Additionally, we further examined the impact of TXNIP on patient survival in a BRAF-mutant advanced melanoma cohort using the TCGA database and found that patients with high TXNIP expression had a significantly longer median survival time than those with low expression (Log Rank, *P* = 0.047), indicating that high expression of TXNIP is a protective prognostic factor for BRAF-mutant advanced melanoma (Supplementary Fig. 5B). Immunofluorescence analysis revealed that TXNIP significantly translocated to mitochondria (labeled by Mito-Tracker) following Vem+DSF treatment (Supplementary Fig. 5C). WB further confirmed a marked increase in TXNIP protein levels in isolated mitochondria (Supplementary Fig. 5D), indicating that Vem+DSF treatment induces mitochondrial localization of TXNIP.

To further verify the key role of TXNIP in DSF+Vem-induced mitochondrial dysfunction, we established a TXNIP-knockout VR cell model using CRISPR-Cas9 technology (Supplementary Fig. 5E). CCK-8 and clone formation assays showed that the inhibitory effect of DSF combination on VR cells was significantly reversed after TXNIP knockout (Fig. 5C, D), indicating the critical role of TXNIP in DSF-induced cytotoxicity. TEM photography showed that the TXNIP knockout canceled the mitochondrial structure damage by the DSF combination (Fig. 5E). Apart from that, the DSF-induced impairments of MMP (Fig. 5F and Supplementary Fig. 5F) and mitochondrial oxidative respiration (Fig. 5G, H and Supplementary Fig. 5G) were restored by TXNIP knockout. Also, DSF failed to provoke excessive mt-ROS in TXNIP knockout cells (Fig. 5I and Supplementary Fig. 5H). In the nude mice model, the Vem+DSF treatment showed no growth inhibition effect on VR cells with TXNIP knockout (Fig. 5J–L).

### TXNIP provokes mitochondrial oxidative stress in vemurafenib-resistant cells via the interaction with TRX2

**Fig. 6:**
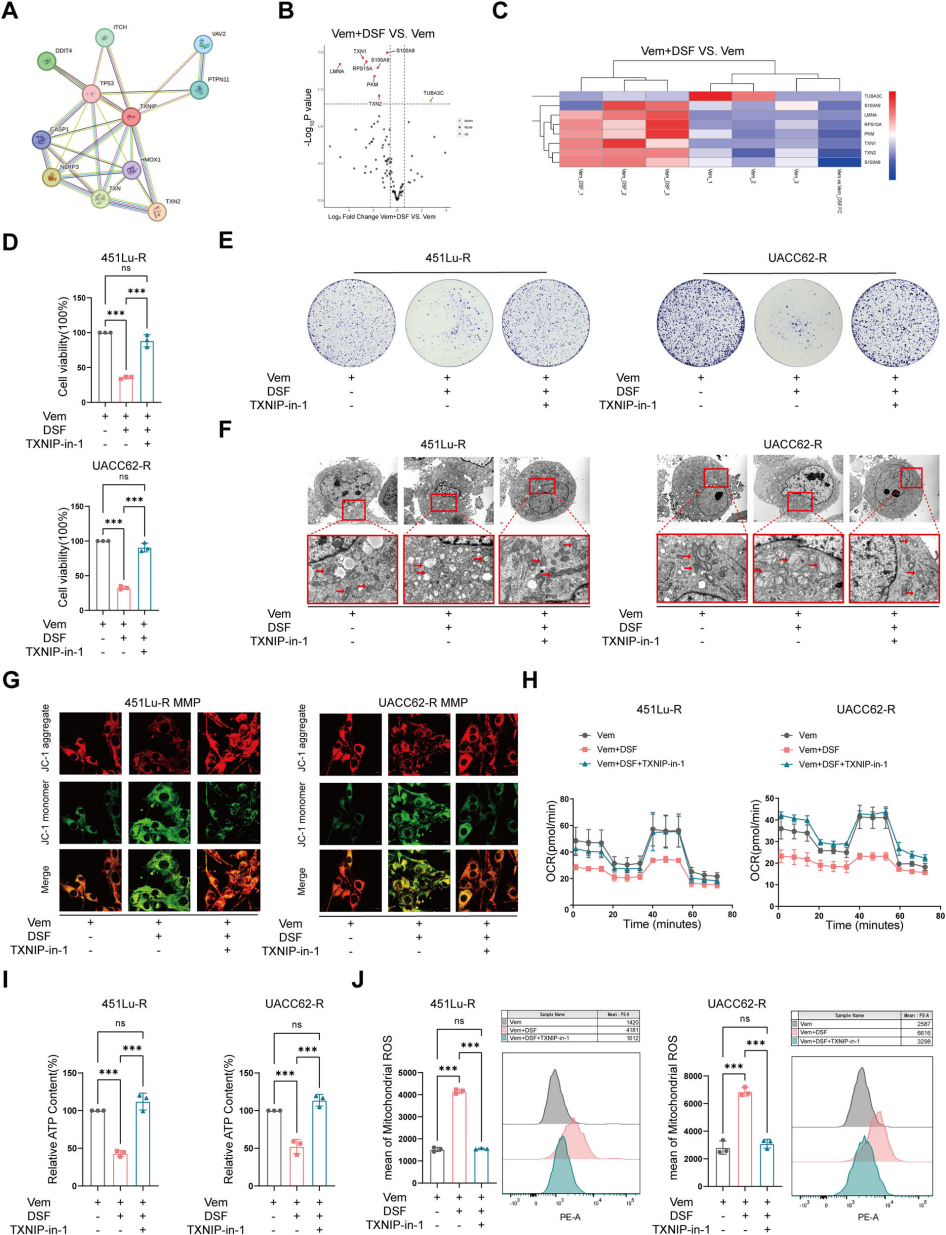
TXNIP provokes mitochondrial oxidative stress in vemurafenib-resistant cells via the interaction with TRX2. **A** Interactome of TXNIP by the STRING database. **B** The volcano plot of proteins interacting with TXNIP by Data-Independent Acquisition (DIA) quantitative proteomics. **C** The heatmap of the proteins interacting with TXNIP by Data-Independent Acquisition (DIA) quantitative proteomics. **D** CCK-8 assay of TXNIP-in-1 pretreated vemurafenib-resistant cells exposed to Vem or Vem+DSF. **E** Colony formation of TXNIP-in-1 pretreated vemurafenib-resistant cells exposed to Vem or Vem+DSF. **F** TEM observation of TXNIP-in-1 pretreated vemurafenib-resistant cells exposed to Vem or Vem+DSF. **G** Immunofluorescence to determine MMP of TXNIP-in-1 pretreated vemurafenib-resistant cells exposed to Vem or Vem+DSF. **H** Seahorse assay of TXNIP-in-1 pretreated vemurafenib-resistant cells exposed to Vem or Vem+DSF. **I** ATP levels of TXNIP-in-1 pretreated vemurafenib-resistant cells exposed to Vem or Vem+DSF. **J** Flow cytometry to detect mt-ROS of TXNIP-in-1 pretreated vemurafenib-resistant cells exposed to Vem or Vem+DSF. Data represent the mean ± SD of triplicate. The differences were analyzed using one-way ANOVA. *P < 0.05, **P < 0.01, and ***P < 0.001. ns non-significant.

Further, we used the STRING database (http://string-db.org) and the DIA quantitative proteomic analysis to clarify the mechanism of TXNIP contributing to DSF-induced cytotoxicity. The interaction network (Fig. 6A) by the STRING database, the volcano plots, and the heat maps by proteomic analysis (Fig. 6B, C) showed proteins with significant changes (*P* < 0.05). Among the candidate proteins, we chose thioredoxin 1 (TXN1/TRX1) and thioredoxin 2 (TXN2/TRX2) as candidate interacting molecules because they are key regulators of intracellular redox balance upon binding to TXNIP [29, 30]. Accordingly, we used TXNIP-IN-1, a recently developed inhibitor of TXNIP-TRX complex formation [31], to determine the effects of the TXNIP-TRX complex. The restoration effects of TXNIP-IN-1 on DSF+Vem-induced growth inhibition were confirmed by CCK-8 assay and clone formation (Fig. 6D, E). Moreover, the pretreatment of TXNIP-IN-1 largely maintained a normal structure and function of mitochondria in cells exposed to Vem+DSF (Fig. 6F–I and Supplementary Fig. 6A, B). Importantly, the TXNIP-IN-1 also protected VR cells from mitochondrial oxidative damage led by Vem+DSF (Fig. 6J and Supplementary Fig. 6C). In addition, CO-IP confirmed an enhanced TXNIP interaction with both TRX1 and TRX2 following DSF+Vem stimulation (Supplementary Fig. 6D, E). To clarify which of TRX1 or TRX2 is more crucial in TXNIP-mediated mitochondrial oxidative damage, we knocked down TRX1 and TRX2, respectively, and found that TRX2 knockdown largely, though not completely, reversed DSF+Vem-induced growth inhibition (Supplementary Fig. 6F, G). On the other hand, TRX1 knockdown (Supplementary Fig. 6H) did not rescue DSF-induced cell death (Supplementary Fig. 6I). In summary, these findings suggest that TXNIP mediates the mitochondrial oxidative damage via the interaction with TRX2.

## Discussion

The combination of BRAF/MEK inhibitors is now the standard of care in advanced melanoma harboring BRAF V600E-mutant due to the improved survival relative to BRAF inhibitor monotherapy [32–34]. Nevertheless, this dual-targeted therapy failed to transfer the clear advantage of objective response rate to better overall survival than immune therapies. The inevitable resistance is believed to be responsible for the majority of progressive diseases [6, 7]. Therefore, novel strategies are still needed to overcome the resistance of MAPK inhibitors. Previously, we demonstrated that the resistance to BRAFi could be reversed by increasing the vulnerability to oxidative stress under BRAFi exposure [35]. Accordingly, the combination of oxidative stress inducers with BRAFi may be a promising strategy to overcome BRAFi resistance.

In the current study, we revealed the synergistic suppressive effect of DSF and vemurafenib on BRAFi-resistant melanoma cells, independent of the suppression of MAPK reactivation. Moreover, we identified that the DSF+Vem combination exerts cytotoxicity by inducing severe intracellular oxidative damage, resulting in significant mitochondrial dysfunction. Importantly, the pretreatment of TTM, a copper chelator, substantially restores the cell death caused by DSF+Vem, suggesting that the combination treatment-induced mitochondrial dysfunction is copper-dependent.

The huge time and cost consumption make new anticancer drug development a great burden worldwide. To solve this problem, drug repurposing is now considered as a promising policy. DSF is a safe and cheap drug that has been used as an alcoholism agent for decades. Recently, it has drawn increasing attention owing to its anticancer activities in different types of cancers, including non-small cell lung cancer (NSCLC), liver cancer, breast cancer, prostate cancer, pancreatic cancer, glioblastoma (GBM), and melanoma [20, 36].

The mechanism of DSF-induced cell death is not fully understood. The known inhibition effects are mainly associated with apoptosis induction as a result of endoplasmic reticulum (ER) stress, autophagy, and oxidative stress [20, 36–38]. The inhibition of NF-kB signaling, proteasome activity, and aldehyde dehydrogenase (ALDH) activity is involved in the above process [39]. However, our findings indicated that, in adaptive BRAFi-resistant melanoma cells, the combination with DSF induces a distinct form of cell death, more likely associated with robust oxidative stress via copper overload rather than apoptosis, necroptosis, or ferroptosis.

The elevation of intracellular oxidative stress by DSF has also been noted in previous studies on melanoma cells [38, 40, 41]. However, the mechanisms underlying this phenomenon have not been explicated. Our study confirmed that mitochondria is an important target of DSF. The swollen shape and abnormal cristae structure observed by transmission electron microscopy, the impaired mitochondrial membrane potential detected by flow cytometry and laser confocal microscopy, as well as the suppression of mitochondrial respiration by the Seahorse assay, demonstrated that DSF leads to severe damage to not only the structure but also essential functions of mitochondria. Whereas the significant rescue effect by TTM suggested that the above changes rely largely on a copper-dependent way. Importantly, although DSF functions as a copper ionophore in this process, modulating FDX1 expression revealed that the inhibitory efficacy of DSF+Vem against resistant cells remained unaffected, suggesting that FDX1-mediated cuproptosis might not constitute the primary pattern of DSF+Vem-induced cell death in BRAF inhibitor-resistant cells. In this study, we identified that TXNIP contributes to DSF-induced mitochondrial dysfunction via interacting with TRX2 and inhibiting its antioxidant function and expression, which has not been reported to date. TRX2 is a member of the thioredoxin antioxidant system, located in mitochondria of most mammalian cells. By suppressing mitochondrial ROS production, TRX2 plays a pivotal role in regulating cellular redox and survival [30]. Our findings shed new light on the mechanism of cell death induced by disulfiram, supporting its further application in BRAF-mutant melanoma.

**Fig. 7:**
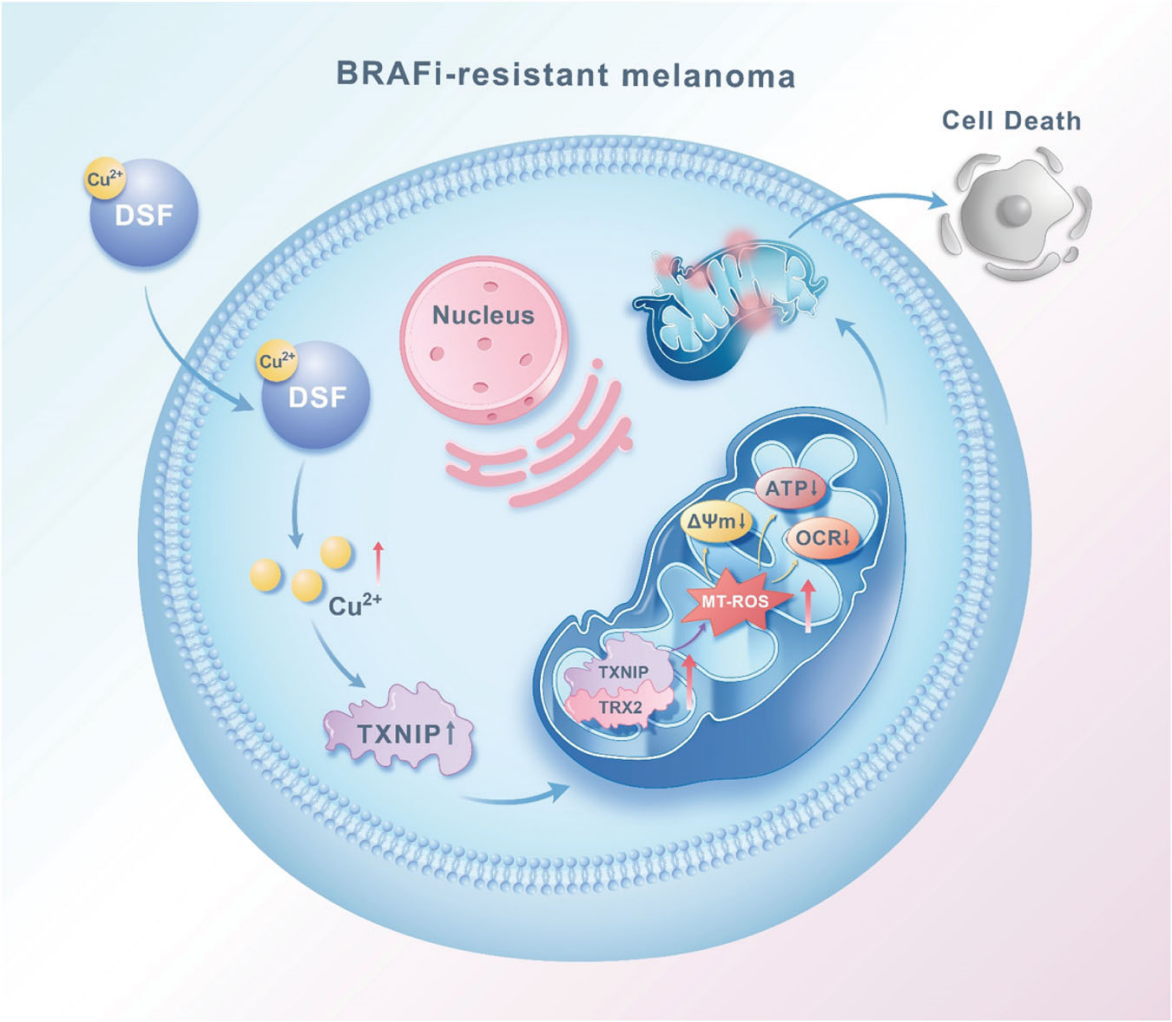
A schematic illustration depicting the mechanism by which disulfiram(DSF) exerts its inhibitory effect on BRAFi-resistant cells by inducing mitochondrial dysfunction. DSF disulfiram, TXNIP thioredoxin-interacting protein, TRX2 thioredoxin 2, MT-ROS mitochondrial reactive oxygen species, OCR, oxygen consumption rate, ΔΨm mitochondrial membrane potential, BRAFi BRAF inhibitors.

Lastly, we summarize the main findings of the current study and present the schematic diagram in Fig. 7.

## Conclusion

Our findings demonstrate that targeting mitochondria with DSF shows significant suppression efficacy in BRAFi-resistant melanoma cells, suggesting the combination of DSF with BRAF inhibitors may be a promising strategy to overcome BRAFi resistance.

## Materials and methods

### Cell culture

The human melanoma cell line 451Lu and the UACC62 cell line were generously provided by Professors Roland Houben and David Schrama from the Dermatology Department of the University Hospital Würzburg, Germany. 451Lu and UACC62 cells were cultured in RPMI 1640 medium supplemented with 10% fetal bovine serum (cat. 10-040-CV, Corning). All the Cells were maintained in a humidified 5% CO_2_ atmosphere at 37 °C.

### Materials

Vemurafenib (HY-12057), trametinib (HY-10999), Z-VAD-FMK (HY-16658B), Ferrostatin-1 (HY-100579), Necrostatin-1 (HY-15760), TXNIP-IN-1 (HY-115688), Mito-TEMPO (HY-112879), and chloroquine (HY-17589A) were purchased from MedChemEXpress (Monmouth Junction, NJ, USA). Tetrathiomolybdate (323446) and Dihydroethidium (D7008) were purchased from Sigma-Aldrich (St. Louis, MO, USA). disulfiram (NSC 190940) S1680 was purchased from Selleck (Houston, USA). Protein A, G & L Agarose Immunoprecipitation Reagents (sc-2003) were purchased from Santa Cruz Biotech (California, USA). MitoSOX™ Mitochondrial Superoxide Indicators (M36008) and eBioscience™ JC-1 Mitochondrial Membrane Potential Dye (65-0851-38) were purchased from Invitrogen (Carlsbad, USA). ATP assay kit (S0026) was purchased from Beyotime (Shanghai, China). Boc-D(OMe)-FMK (ALX-260-071-M005) was purchased from ENZO (New York, USA). Hanks’ balanced salt solution (HBSS, H8264) was purchased from Merck Millipore (Billerica, MA, USA).

For the treatment of cells, we chose disulfiram 1.5 μM, vemurafenib 5 μM, and trametinib 2 nM, respectively.

### Establishment of BRAFi-resistant cells

The parental 451Lu and UACC62 cells (referred to as 451Lu-P and UACC62-P) were plated at a density of 5 × 10^3^ cells per well in 96-well plates and continuously exposed to vemurafenib with incrementally elevated concentration (0.2, 0.5, 1, 2, and 5 μM). The sensitivity of melanoma cells to vemurafenib (HY-12057, MCE) was assessed by determining the half-maximal inhibitory concentration (IC50). After 16 weeks, two BRAFi-resistant cell lines, designated as vemurafenib-resistant 451Lu cells (451Lu-R) and vemurafenib-resistant UACC62 cells (UACC62-R), were successfully established and maintained in the medium with vemurafenib 5 μM.

### CRISPR-Cas9

For knocking out TXNIP, a lentivirus constructed using the CRISPR-Cas9 system targeting TXNIP was obtained from Beijing Tsingke Biotechnology Co., Ltd. (China). The specific targeting sequence for the knockout of TXNIP is as follows: TCTCTTGAGTTGGCTGGCTC. The cells were infected using this lentivirus according to the manufacturer’s protocol. After 72 h of transfection, the cells were selected for stable knockouts in RPMI 1640 culture medium supplemented with puromycin (10 µg/mL) (Beyotime, China). The efficiency of the gene knockout was verified through WB analysis.

### siRNA transfection and plasmid vectors

siRNA targeting FDX1, TRX1, and TRX2, as well as the FDX1 plasmid, were purchased from Beijing Tsingke Biotechnology Co., Ltd. (China). Plasmid and siRNA transfection were performed using the Lipofectamine 3000 Transfection Reagent Kit (Catalog Number: L3000-015, Invitrogen, Carlsbad, USA) according to the manufacturer’s recommended protocols. The sequences of the siRNAs used are as follows:si-FDX1: 5′ - CAUGCUCGAUCUGGCAUAU - 3′; si-TRX1: 5′ - CAUUAAUGAAUUAGUCUAA - 3′; si-TRX2: 5′ - GGUCAACAGUGAGACACCA - 3′.

### Western blotting (WB)

In brief, harvested cells were rinsed three times with phosphate-buffered saline (PBS). Cell lysates were prepared using a lysis buffer, followed by centrifugation at 12,000 × *g* for 15 min at 4 °C. Protein concentrations were determined using the BCA Protein Assay Kit (catalog number E-BC-K318-M, ElabScience). Equivalent amounts of protein samples were separated using SDS-PAGE and transferred to polyvinylidene fluoride (PVDF) membranes. The membranes were blocked for one hour in 5% non-fat dry milk and incubated with the primary antibodies overnight at 4 °C. After TBST washing, membranes were incubated using horseradish peroxidase (HRP)-conjugated secondary antibodies at room temperature for 1 h. The immunoreactive bands were visualized using an Enhanced Chemiluminescence (ECL) Protein Detection System from Thermo Scientific. The antibodies used in this investigation are listed below: Caspase 3/p17/p19 (1:1000, Proteintech, cat. 19677-1-AP), TXNIP (1:1000, Abcam, cat. ab188865), Thioredoxin 2 (1:1000, Cell Signaling Technology, cat. 14907), ERK1/2 (1:1000, Cell Signaling Technology, cat. 4695), Phospho ERK1/2 (Thr202/Tyr204) (1:1000, Cell Signaling Technology, cat. 9101), Thioredoxin (1:1000, Proteintech, cat. 14999-1-AP), TOM20 (1:5000, Proteintech, cat. 11802-1-AP), ATP7A (1:1000, Abcam, cat. ab308524), ATP7B (1:1000, Abcam, cat. ab124973), SLC31A1 (1:1000, Proteintech, cat. 67221-1-IG), DLAT (1:1000, Proteintech, cat. 13426-1-AP), LIAS (1:1000, Proteintech, cat. 11577-1-AP), FDX1 (1:1000, Proteintech, cat. 12592-1-AP), Tubulin (1:5000, Proteintech, cat.11224-1-AP), goat anti-mouse IgG (1:5000, Proteintech, cat.SA00004-1) and goat anti-rabbit IgG (1:5000, Proteintech, cat.SA00001-2).

### Cell viability assay

451Lu-R and UACC62-R cells were cultured at a density of 5000 cells per well in a 96-well plate and treated with the indicated reagents. After treatment, replace the culture medium with the diluted CCK-8 reagent (cat. GK10001) at a dilution factor of 1:10 and incubate for 1 h at 37 °C with 5% CO_2_. The Model 680 microplate reader (Bio-Rad Laboratories, Hercules, CA, USA) was employed to measure the absorbance at 450 nm.

### Mitochondrial reactive oxygen species (mt-ROS) assays

To perform the flow cytometry, cells were collected and rinsed twice using PBS pre-equilibrated to 37 °C. Subsequently, the cells were incubated with a 5 µM solution of MitoSOX™ Red (cat. M36008, Carlsbad, USA, Invitrogen) for 20 min at 37 °C, protected from light. Then the cells were washed twice with pre-warmed PBS, detached with trypsin, and resuspended in PBS, and analyzed on BD LSRFortessa™ Flow Cytometer (BD Biosciences, Franklin Lakes, NJ, USA). To perform the confocal microscopy imaging, cells were seeded into confocal dishes at a density of 1 × 10^5^ per well. 25 µM MitoSOX™ Red (cat. M36008, Carlsbad, USA, Invitrogen) was introduced into 1 mL of fresh culture medium per well and incubated at 37 °C for 20 min. Unbound dye was removed by gently washing with HBSS. Visualization of the stained cells was performed using a Zeiss LSM880 confocal laser scanning microscope (Zeiss, Oberkochen, Germany).

### Colony formation assay

451Lu-R and UACC62-R melanoma cells were seeded in 6-well plates with a density of 3000 cells per well and incubated for two weeks. Subsequently, the cells were washed with phosphate-buffered saline (PBS), fixed with a 5% methanol solution, and stained with 1% crystal violet for a minimum of 30 min. Following staining, enumerate the visible colonies.

### Co-immunoprecipitation (Co-IP)

Protein lysates were prepared in pre-cooled immunoprecipitation (IP) lysis buffer, followed by a 20-min incubation at 4 °C. Pellet the cellular debris by centrifugation at 12,000 × *g* for 15 min at 4 °C. The protein extracts were incubated with 1–10 μL of the primary antibody (TXNIP(D5F3E) (1:50, Cell Signaling Technology, cat. 14715)) or an appropriate control IgG (Proteintech, #30000-0-AP) for 3 h at 4 °C. 40 μL of Protein A/G PLUS-Agarose (Santa Cruz, cat. sc-2003) was resuspended and added to the lysate, and then incubated overnight at 4 °C. After that, the beads were washed with cold PBS four times, and the precipitate was resuspended in 1× loading buffer and boiled for 10 min. Finally, the boiled samples were analyzed by WB.

### Mitochondrial membrane potential (MMP)

The JC-1 fluorescent probe was used to assess the change of MMP. 1 mL of the JC-1 working solution was added to the cells and incubated for 30 min at 37 °C. After that, the supernatant was aspirated, and the cells were rinsed twice using HBSS. Subsequently, the cells with 400 μL of PBS were observed using a confocal laser scanning microscope.

To perform the flow cytometry, the vemurafenib-resistant cells were treated with 5 μM MitoSOX™ Red and incubated at 37 °C in a light-protected environment for 20 min. Following incubation, the cells were washed twice with HBSS, dissociated using trypsin, and resuspended in 400 μL of PBS. The cells were analyzed on a BD LSRFortessa™ Flow Cytometer.

### Immunofluorescence

The vemurafenib-resistant cells were cultured in a laser confocal dish, washed with HBSS, fixed with 4% paraformaldehyde for 15 min, and permeabilized with 0.5% Triton X-100 for 10 min. Cells were then blocked with goat serum (Gibco, USA) for 30 min and incubated with the primary antibody at 4 °C overnight, followed by incubation with the secondary antibody at room temperature for 1 h. The stained cells were observed with the Zeiss LSM880 confocal laser scanning microscope (Zeiss, Germany). The primary and secondary antibodies were rabbit anti-TXNIP (1:50, Proteintech, cat. 18243-1-AP), MitoTracker^TM^ Red CMXROS (1:2000, Life technologies, cat. M7512), goat anti-rabbit IgG H&L (FITC) (1:150, Zhuangzhibio, cat. EK023), and goat anti-mouse IgG H&L (Cy3) (1:150, Zhuangzhibio, cat. EK012).

### Transmission electron microscope (TEM) analysis

For each indicated group, 5 × 10^6^ cells were collected, fixed with 2.5% glutaraldehyde and proceeded to Chengdu Lilai Technology Co., Ltd. (Chengdu, China) for subsequent detection steps: (1) Fixation: fixation with 1% osmium tetroxide; (2) Dehydration: Incremental dehydration using acetone at concentration gradients of 30% to 100% (with three changes at 100% concentration); (3) Infiltration: infiltration with mixtures of the dehydrating agent and Epon-812 embedding agent at volume ratios of 3:1, 1:1, and 1:3; (4) Embedding: Complete embedding using pure Epon-812 embedding agent; (5) Ultrathin sectioning: 60~90 nm ultrathin sections were produced with an ultramicrotome and transferred to copper grids; (6) Staining: Initial staining with uranyl acetate for 10–15 min, followed by lead citrate staining for 1–2 min, performed at room temperature; (7) Image acquisition: the JEM-1400FLASH transmission electron microscope from Japan Electronics Corporation (JEOL) was used to capture images on the copper grids.

### Seahorse assays

Vemurafenib-resistant cells were placed in the microplate designed for Seahorse XF analysis to reach a confluence of 70–90%. After 24-h incubation, cells were treated with the indicated drugs. The subsequent assays for oxygen consumption rate (OCR) and extracellular acidification rate (ECAR) were performed by the Biochemistry Laboratory, Fourth Military Medical University. The drugs used for OCR measurement included: 1 µM Oligomycin, 1 µM FCCP, and 2 µM Rotenone & Antimycin A. For ECAR measurement, the drugs used included 100 mM glucose, 10 µM Oligomycin, and 500 µM 2-deoxy-D-glucose (2-DG). Data were analyzed using Seahorse Wave software in conjunction with GraphPad Prism.

### RNA sequencing (RNA-seq)

Extraction of total RNA was performed using TRIzol reagent. Following RNA extraction, the RNA sample processing and sequencing analyses were conducted by Maiwei Metabolism(Wuhan, China).

### ATP level and luciferase assay

Cells were lysed with ATP lysis buffer on ice for 15 min and centrifuged at 12,000 × *g* for 5 min at 4 °C to pellet cellular debris. The supernatant obtained was subjected to ATP quantification using the ATP Detection Kit (cat. S0026, Beyotime, China) according to the manufacturer’s instructions.

### Data-independent acquisition (DIA) quantitative proteomic analysis

10^7^ 451Lu-R cells were collected from each indicated group using the TXNIP antibody (cat.14715, Cell Signaling and Technology). Then, the samples were submitted to Novogene (Beijing, China) for DIA quantitative proteomic analysis.

### The Cancer Genome Atlas (TCGA) database

Consult the Cancer Genome Atlas(TCGA) database by visiting http://www.cbioportal.org to determine the effect of TXNIP on survival in melanoma patients carrying BRAF mutations. This repository offers comprehensive transcriptional data and follow-up details for individuals afflicted with BRAF-mutant melanoma.

### Animal experiments

The animal experiments were approved by the Animal Protection and Utilization Committee of the animal facility at the Fourth Military Medical University. Six-week-old female BALB/c nude mice were used in the experiment. Tumor growth of mice was measured every two days by quantifying tumor length (L) and width (W) (tumor volume = L × W^2^ × 0.5). 1 × 10^7^ cells were injected subcutaneously into the BALB/c nude mice. Upon reaching a tumor volume of 50 mm^3^, the mice were randomly divided into different groups and given the indicated treatments. For the treatment of mice, we chose vemurafenib (30 mg/kg), trametinib (1 mg/kg), DSF (60 mg/kg), and TTM (0.3 mg/kg), respectively. For further analysis, subcutaneously grown tumors were removed surgically, weighed, and photographed.

### Statistical analysis

The data are presented as the mean ± standard error and were analyzed statistically using GraphPad Prism 9.0. To determine the statistical significance between two groups, two-tailed Student’s *t*-tests were used. For the comparison of differences across multiple groups, one-way ANOVA was applied. Furthermore, the correlation between the measured variables was evaluated using Pearson’s correlation analysis. Unless otherwise indicated, each experiment was performed a minimum of three times to ensure reproducibility and reliability of the results. Statistical significance was defined as a *P*-value of less than 0.05.

## Supplementary information

Supplementary Figures and Legends

Western blotting

## Data Availability

Upon reasonable request, the corresponding author will provide the datasets used and/or analyzed during the current study.

## References

[1] Davies H, Bignell GR, Cox C, Stephens P, Edkins S, Clegg S, et al. Mutations of the BRAF gene in human cancer. Nature. 2002;417:949–54. 12068308 10.1038/nature00766

[2] Larkin J, Lao CD, Urba WJ, Mcdermott DF, Horak C, Jiang J, et al. Efficacy and safety of nivolumab in patients with BRAF V600 mutant and BRAF wild-type advanced melanoma: a pooled analysis of 4 clinical trials. JAMA Oncol. 2015;1:433–40. 26181250 10.1001/jamaoncol.2015.1184

[3] Chapman PB, Hauschild A, Robert C, Haanen JB, Ascierto P, Larkin J, et al. Improved survival with vemurafenib in melanoma with BRAF V600E mutation. N Engl J Med. 2011;364:2507–16. 21639808 10.1056/NEJMoa1103782 PMC3549296

[4] Hauschild A, Ascierto PA, Schadendorf D, Grob JJ, Ribas A, Kiecker F, et al. Long-term outcomes in patients with BRAF V600-mutant metastatic melanoma receiving dabrafenib monotherapy: analysis from phase 2 and 3 clinical trials. Eur J Cancer. 2020;125:114–20. 31864178 10.1016/j.ejca.2019.10.033 PMC8073226

[5] Ascierto PA, Dreno B, Larkin J, Ribas A, Liszkay G, Maio M, et al. 5-Year outcomes with cobimetinib plus vemurafenib in BRAFV600 mutation-positive advanced melanoma: extended follow-up of the coBRIM study. Clin Cancer Res. 2021;27:5225–35. 34158360 10.1158/1078-0432.CCR-21-0809 PMC9401485

[6] Sosman JA, Kim KB, Schuchter L, Gonzalez R, Pavlick AC, Weber JS, et al. Survival in BRAF V600-mutant advanced melanoma treated with vemurafenib. N Engl J Med. 2012;366:707–14. 22356324 10.1056/NEJMoa1112302 PMC3724515

[7] Ascierto PA, Dummer R, Gogas HJ, Flaherty KT, Arance A, Mandala M, et al. Update on tolerability and overall survival in COLUMBUS: landmark analysis of a randomised phase 3 trial of encorafenib plus binimetinib vs vemurafenib or encorafenib in patients with BRAF V600-mutant melanoma. Eur J Cancer. 2020;126:33–44. 31901705 10.1016/j.ejca.2019.11.016

[8] Rizos H, Menzies AM, Pupo GM, Carlino MS, Fung C, Hyman J, et al. BRAF inhibitor resistance mechanisms in metastatic melanoma: spectrum and clinical impact. Clin Cancer Res. 2014;20:1965–77. 24463458 10.1158/1078-0432.CCR-13-3122

[9] Van Allen EM, Wagle N, Sucker A, Treacy DJ, Johannessen CM, Goetz EM, et al. The genetic landscape of clinical resistance to RAF inhibition in metastatic melanoma. Cancer Discov. 2014;4:94–109. 24265153 10.1158/2159-8290.CD-13-0617 PMC3947264

[10] Robert C, Grob JJ, Stroyakovskiy D, Karaszewska B, Hauschild A, Levchenko E, et al. Five-year outcomes with dabrafenib plus trametinib in metastatic melanoma. N Engl J Med. 2019;381:626–36. 31166680 10.1056/NEJMoa1904059

[11] Ferraz LS, Costa R, Costa C, Ribeiro C, Arruda DC, Maria-Engler SS, et al. Targeting mitochondria in melanoma: interplay between MAPK signaling pathway and mitochondrial dynamics. Biochem Pharm. 2020;178:114104. 32562785 10.1016/j.bcp.2020.114104

[12] Najem A, Krayem M, Sabbah S, Pesetti M, Journe F, Awada A, et al. Targeting prohibitins to inhibit melanoma growth and overcome resistance to targeted therapies. Cells. 2023;12:14. 10.3390/cells12141855 PMC10378173 37508519

[13] Figarola JL, Singhal J, Singhal S, Kusari J, Riggs A. Bioenergetic modulation with the mitochondria uncouplers SR4 and niclosamide prevents proliferation and growth of treatment-naive and vemurafenib-resistant melanomas. Oncotarget. 2018;9:36945–65. 30651927 10.18632/oncotarget.26421 PMC6319337

[14] Carpenter EL, Chagani S, Nelson D, Cassidy PB, Laws M, Ganguli-Indra G, et al. Mitochondrial complex I inhibitor deguelin induces metabolic reprogramming and sensitizes vemurafenib-resistant BRAF(V600E) mutation bearing metastatic melanoma cells. Mol Carcinog. 2019;58:1680–90. 31211467 10.1002/mc.23068 PMC6692247

[15] Vashisht GY, Gammon S, Prasad R, Knighton B, Pisaneschi F, Roszik J, et al. A novel mitochondrial inhibitor blocks MAPK pathway and overcomes MAPK inhibitor resistance in melanoma. Clin Cancer Res. 2019;25:6429–42. 31439581 10.1158/1078-0432.CCR-19-0836 PMC6825560

[16] Ruiz LM, Libedinsky A, Elorza AA. Role of copper on mitochondrial function and metabolism. Front Mol Biosci. 2021;8:711227. 34504870 10.3389/fmolb.2021.711227 PMC8421569

[17] Tsvetkov P, Coy S, Petrova B, Dreishpoon M, Verma A, Abdusamad M, et al. Copper induces cell death by targeting lipoylated TCA cycle proteins. Science. 2022;375:1254–61. 35298263 10.1126/science.abf0529 PMC9273333

[18] Liu Y, Guan X, Wang M, Wang N, Chen Y, Li B, et al. Disulfiram/Copper induces antitumor activity against gastric cancer via the ROS/MAPK and NPL4 pathways. Bioengineered. 2022;13:6579–89. 35290151 10.1080/21655979.2022.2038434 PMC9278967

[19] Xie J, Liu J, Zhao M, Li X, Wang Y, Zhao Y, et al. Disulfiram/Cu kills and sensitizes BRAF-mutant thyroid cancer cells to BRAF kinase inhibitor by ROS-dependently relieving feedback activation of MAPK/ERK and PI3K/AKT p. Int J Mol Sci. 2023;24:4. 10.3390/ijms24043418 PMC9968072 36834830

[20] Zeng M, Wu B, Wei W, Jiang Z, Li P, Quan Y, et al. Disulfiram: a novel repurposed drug for cancer therapy. Chin Med J. 2024;137:1389–98. 38275022 10.1097/CM9.0000000000002909 PMC11188872

[21] Allensworth JL, Evans MK, Bertucci F, Aldrich AJ, Festa RA, Finetti P, et al. Disulfiram (DSF) acts as a copper ionophore to induce copper-dependent oxidative stress and mediate anti-tumor efficacy in inflammatory breast cancer. Mol Oncol. 2015;9:1155–68. 25769405 10.1016/j.molonc.2015.02.007 PMC4493866

[22] Shim D, Han J. Coordination chemistry of mitochondrial copper metalloenzymes: exploring implications for copper dyshomeostasis in cell death. BMB Rep. 2023;56:575–83. 37915136 10.5483/BMBRep.2023-0172 PMC10689082

[23] Deng J, Zhuang H, Shao S, Zeng X, Xue P, Bai T, et al. Mitochondrial-targeted copper delivery for cuproptosis-based synergistic cancer therapy. Adv Health Mater. 2024;13:e2304522. 10.1002/adhm.202304522 38530073

[24] Chen L, Min J, Wang F. Copper homeostasis and cuproptosis in health and disease. Signal Transduct Target Ther. 2022;7:378. 36414625 10.1038/s41392-022-01229-y PMC9681860

[25] Ruiz LM, Jensen EL, Rossel Y, Puas GI, Gonzalez-Ibanez AM, Bustos RI, et al. Non-cytotoxic copper overload boosts mitochondrial energy metabolism to modulate cell proliferation and differentiation in the human erythroleukemic cell line K562. Mitochondrion. 2016;29:18–30. 27094959 10.1016/j.mito.2016.04.005

[26] Jensen EL, Gonzalez-Ibanez AM, Mendoza P, Ruiz LM, Riedel CA, Simon F, et al. Copper deficiency-induced anemia is caused by a mitochondrial metabolic reprograming in erythropoietic cells. Metallomics. 2019;11:282–90. 30358789 10.1039/c8mt00224j

[27] Dreishpoon MB, Bick NR, Petrova B, Warui DM, Cameron A, Booker SJ, et al. FDX1 regulates cellular protein lipoylation through direct binding to LIAS. J Biol Chem. 2023;299:105046. 37453661 10.1016/j.jbc.2023.105046 PMC10462841

[28] Deng J, Pan T, Liu Z, Mccarthy C, Vicencio JM, Cao L, et al. The role of TXNIP in cancer: a fine balance between redox, metabolic, and immunological tumor control. Br J Cancer. 2023;129:1877–92. 37794178 10.1038/s41416-023-02442-4 PMC10703902

[29] He L, He T, Farrar S, Ji L, Liu T, Ma X. Antioxidants maintain cellular redox homeostasis by elimination of reactive oxygen species. Cell Physiol Biochem. 2017;44:532–53. 29145191 10.1159/000485089

[30] Yang B, Lin Y, Huang Y, Shen YQ, Chen Q. Thioredoxin (Trx): a redox target and modulator of cellular senescence and aging-related diseases. Redox Biol. 2024;70:103032. 38232457 10.1016/j.redox.2024.103032 PMC10827563

[31] Singh LP, Devi TS. Potential combination drug therapy to prevent redox stress and mitophagy dysregulation in retinal muller cells under high glucose conditions: implications for diabetic retinopathy. Diseases. 2021;9:4. 10.3390/diseases9040091 PMC8700204 34940029

[32] Zhong J, Yan W, Wang C, Liu W, Lin X, Zou Z, et al. BRAF inhibitor resistance in melanoma: mechanisms and alternative therapeutic strategies. Curr Treat Options Oncol. 2022;23:1503–21. 36181568 10.1007/s11864-022-01006-7 PMC9596525

[33] Rubanov A, Berico P, Hernando E. Epigenetic mechanisms underlying melanoma resistance to immune and targeted therapies. Cancers. 2022;14:23. 10.3390/cancers14235858 PMC9738385 36497341

[34] Tangella LP, Clark ME, Gray ES. Resistance mechanisms to targeted therapy in BRAF-mutant melanoma - a mini review. Biochim Biophys Acta Gen Subj. 2021;1865:129736. 32956754 10.1016/j.bbagen.2020.129736

[35] Guo S, Yue Q, Wang S, Wang H, Ye Z, Zhang W, et al. Sestrin2 contributes to BRAF inhibitor resistance via reducing redox vulnerability of melanoma cells. J Dermatol Sci. 2023;109:52–60. 36858850 10.1016/j.jdermsci.2022.12.007

[36] Lu C, Li X, Ren Y, Zhang X. Disulfiram: a novel repurposed drug for cancer therapy. Cancer Chemother Pharm. 2021;87:159–72. 10.1007/s00280-020-04216-8 33426580

[37] Cen D, Brayton D, Shahandeh B, Meyskens FJ, Farmer PJ. Disulfiram facilitates intracellular Cu uptake and induces apoptosis in human melanoma cells. J Med Chem. 2004;47:6914–20. 15615540 10.1021/jm049568z

[38] Cen D, Gonzalez RI, Buckmeier JA, Kahlon RS, Tohidian NB, Meyskens FJ. Disulfiram induces apoptosis in human melanoma cells: a redox-related process. Mol Cancer Ther. 2002;1:197–204. 12467214

[39] Li Y, Wang LH, Zhang HT, Wang YT, Liu S, Zhou WL, et al. Disulfiram combined with copper inhibits metastasis and epithelial-mesenchymal transition in hepatocellular carcinoma through the NF-kappaB and TGF-beta pathways. J Cell Mol Med. 2018;22:439–51. 29148232 10.1111/jcmm.13334 PMC5742719

[40] Fontes SS, Nogueira ML, Dias RB, Rocha C, Soares M, Vannier-Santos MA, et al. Combination therapy of curcumin and disulfiram synergistically inhibits the growth of B16-F10 melanoma cells by inducing oxidative stress. Biomolecules. 2022;12:11. 10.3390/biom12111600 PMC9687191 36358950

[41] Gao Y, Cai X, Zou W, Tang X, Jiang L, Hao J, et al. Self-supplying Cu(2+) and H(2)O(2) synergistically enhancing disulfiram-mediated melanoma chemotherapy. RSC Adv. 2024;14:13180–9. 38655468 10.1039/d4ra01075b PMC11036371

