# Supplementary material for: Exploiting mitochondrial dysfunction to overcome BRAF inhibitor resistance in advanced melanoma: the role of disulfiram as a copper ionophore
Source: Cell Death Dis. 2025 Jul 1;16(1):482. doi: 10.1038/s41419-025-07766-y (PMC12216038; doi:10.1038/s41419-025-07766-y)
Supplement: Supplementary file 1 — Supplementary Figures and Legends [file 41419_2025_7766_MOESM1_ESM.docx]

**
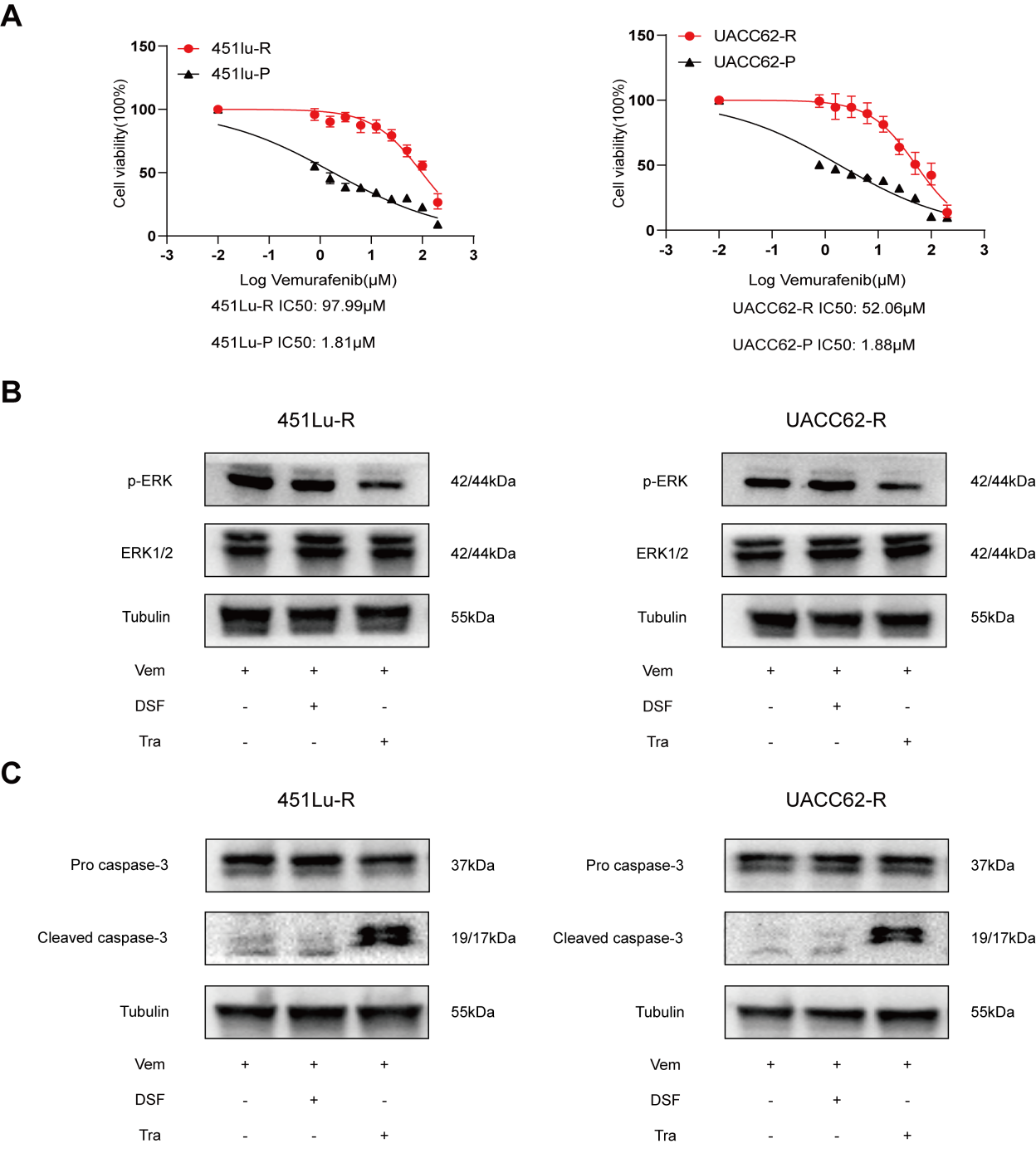
Supplementary Fig. 1 The change of MAPK activation and apoptosis-related proteins.** **A** IC50 assay of 451Lu parental (451Lu-P), 451Lu vemurafenib-resistant (451Lu-R), UACC62 parental (UACC62-P), UACC62 vemurafenib-resistant (UACC62-R) melanoma cells. **B** WB to detect the extracellular signal-regulated kinase (ERK) and phosphorylated ERK (p-ERK) of vemurafenib-resistant cells treated with Vem/ Vem+DSF/ Vem+Tra for 48 hours. **C** WB to detect the cleaved caspase-3 and pro caspase-3 protein expression of vemurafenib-resistant cells treated with Vem/ Vem+DSF/ Vem+Tra for 48 hours.

**
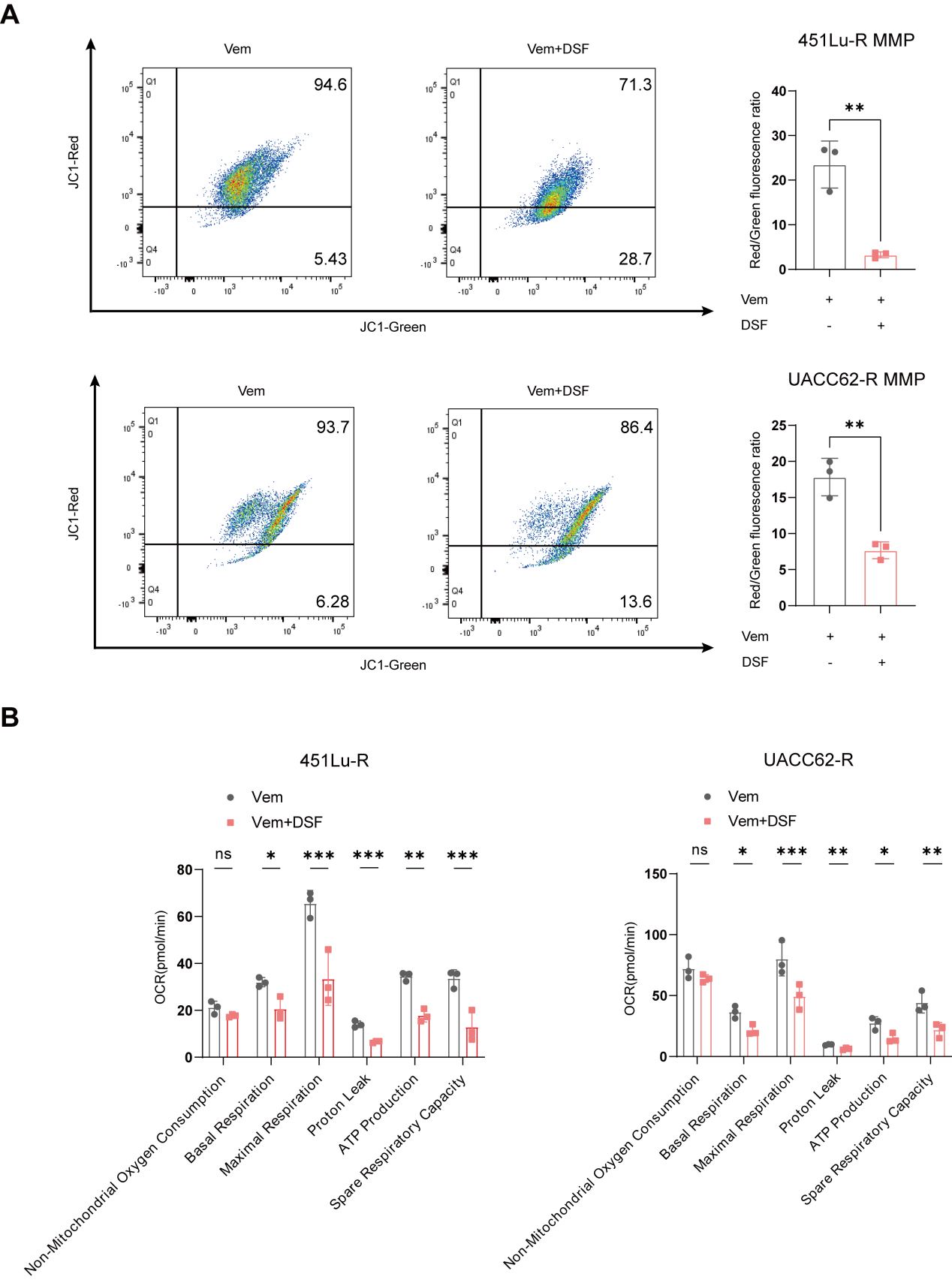
Supplementary Fig. 2 DSF reverses the resistance to vemurafenib by inducing mitochondrial dysfunction. A** Flow cytometry analysis of MMP in vemurafenib-resistant cells with indicated treatment. **B** Seahorse analysis of mitochondrial oxidative phosphorylation in vemurafenib-resistant cells with indicated treatment.

Data represent the mean ± SD of triplicates. *P-*value was calculated by two-­tailed Student’s t-­test. **P* < 0.05, ***P* < 0.01, and ****P* < 0.001. *ns* non-significant.


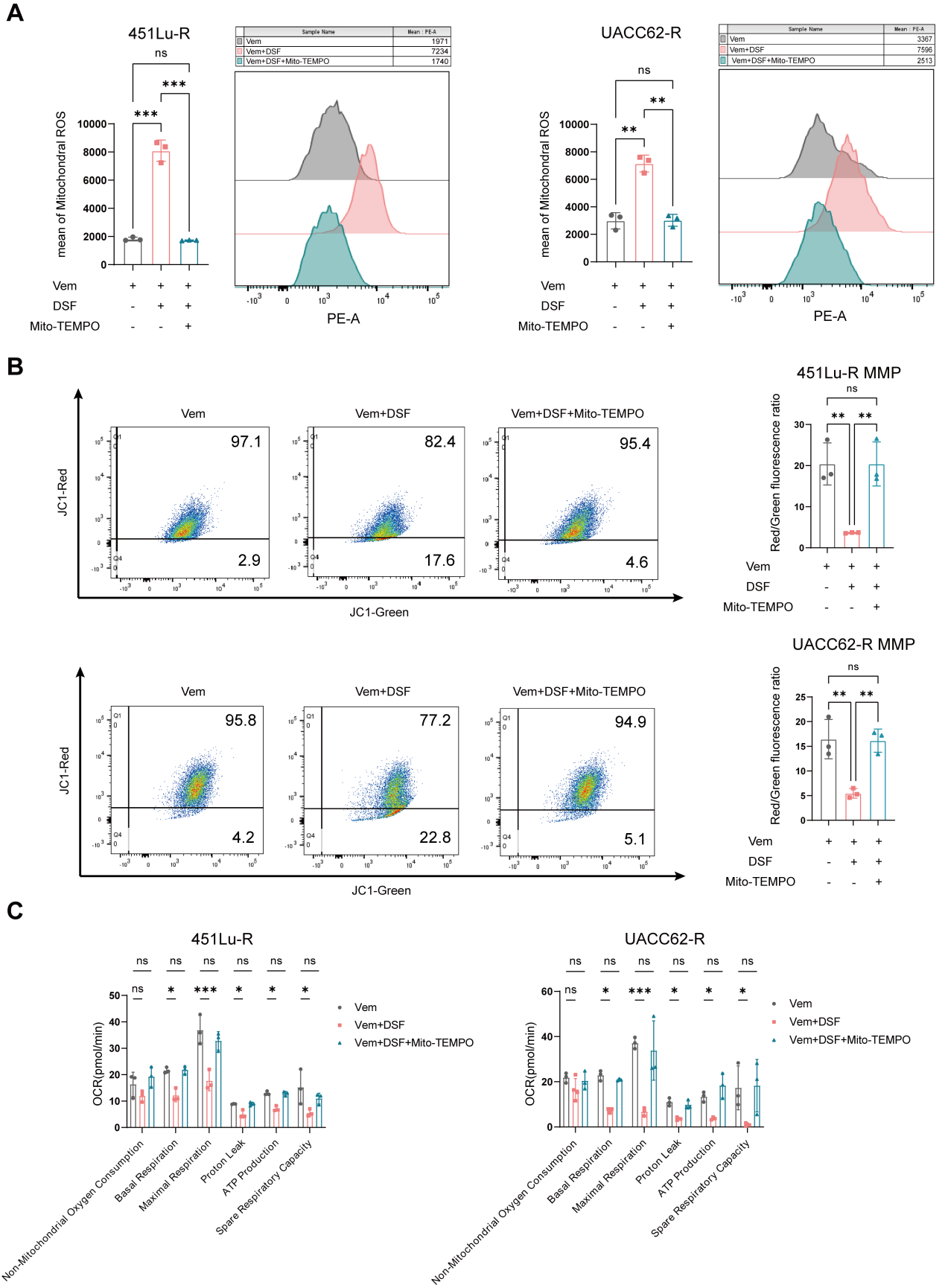


**Supplementary Fig. 3 Clearance of mitochondrial ROS reverses the inhibition effect of DSF. A** Flow cytometry analysis of mt-ROS in vemurafenib-resistant cells exposed to the indicated treatments following the pretreatment with or without Mito-TEMPO. **B** Flow cytometry staining of MMP levels in vemurafenib-resistant cells exposed to the indicated treatments following the pretreatment with or without Mito-TEMPO. **C** Seahorse analysis in vemurafenib-resistant cells exposed to the indicated treatments following the pretreatment with or without Mito-TEMPO.

Data represent the mean ± SD of triplicates. The differences were analyzed using one-way ANOVA. **P* < 0.05, ***P* < 0.01, and ****P* < 0.001. *ns* non-significant.

**
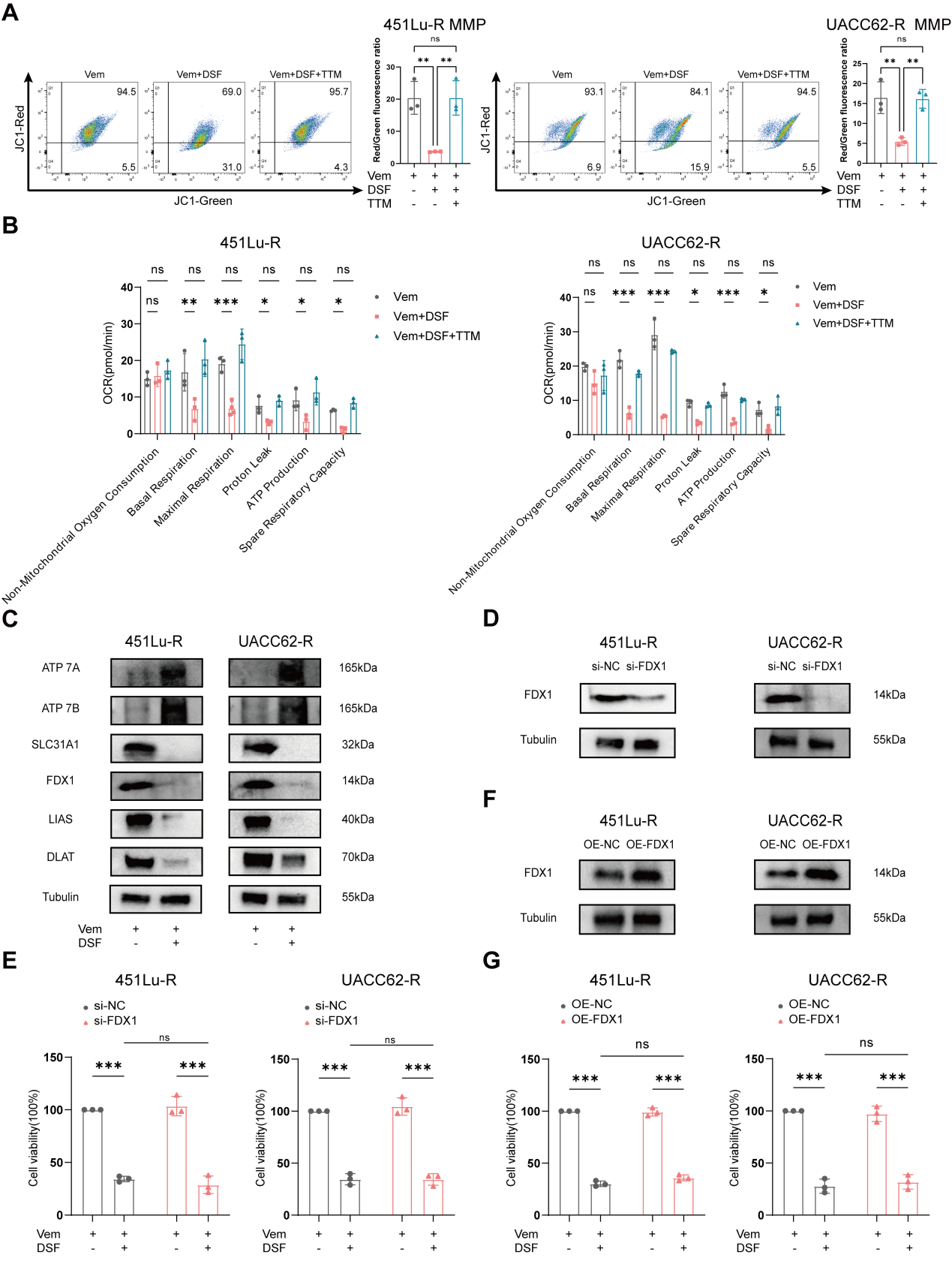
Supplementary Fig. 4 Depletion of copper rescues the DSF-induced mitochondrial dysfunction by reversing the oxidative damage. A** Flow cytometry analysis of MMP levels in vemurafenib-resistant cells exposed to the indicated treatments. **B** Seahorse assay of vemurafenib-resistant cells exposed to the indicated treatments. **C** WB to detect the expression levels of ATP7A, ATP7B, SLC31A1, PDX1, LIAS, and DLAT in vemurafenib-resistant cells treated with Vem alone or in combination with Vem+DSF for 48 hours. **D** WB analysis of the efficacy of FDX1 knockdown. **E** CCK-8 assay of vemurafenib-resistant cells with FDX1 knockdown exposed to Vem+DSF or Vem. **F** WB analysis of the efficacy of FDX1 overexpression. **G** CCK-8 assay of vemurafenib-resistant cells with FDX1 overexpression exposed to Vem+DSF or Vem.

Data represent the mean ± SD of triplicates. The differences were analyzed using one-way ANOVA. **P* < 0.05, ***P* < 0.01, and ****P* < 0.001. *ns* non-significant, si-NC siRNA negative control, si-FDX1 siRNA targeting FDX1, OE-NC overexpression negative control, OE-FDX1 overexpression of FDX1.


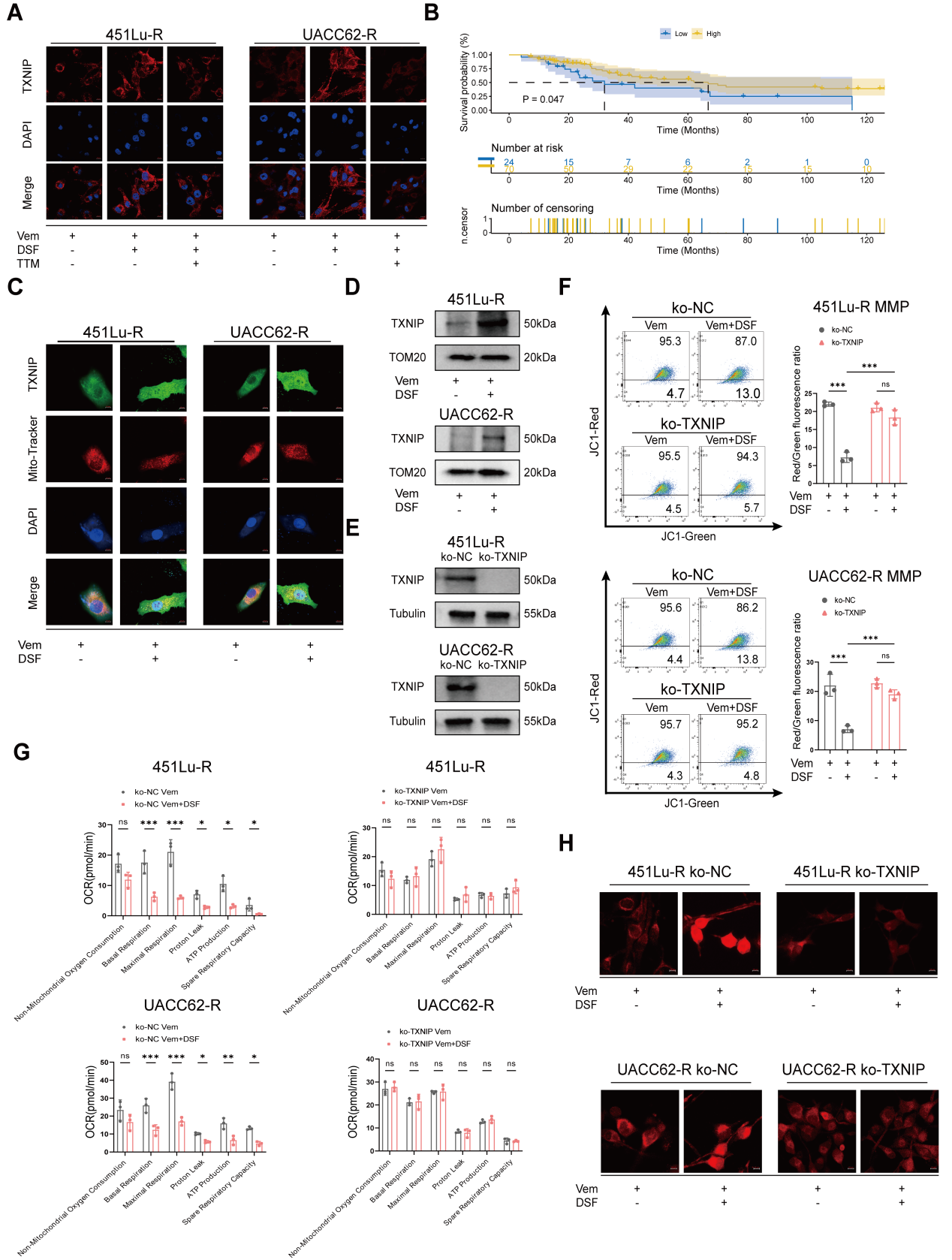


**Supplementary Fig. 5 TXNIP is responsible for DSF-induced mitochondrial dysfunction. A** Immunofluorescence staining of TXNIP expression (red) in vemurafenib-resistant cells exposed to the indicated treatments. **B** The Kaplan-Meier analysis based on the Cancer Genome Atlas (TCGA) database for BRAF-mutated melanoma patients with high or low expression of TXNIP. **C** Immunofluorescence staining of TXNIP (green) and Mito-Tracker (red) in vemurafenib-resistant cells exposed to the indicated treatment. **D** WB analysis of TXNIP expression in mitochondria isolated from vemurafenib-resistant cells exposed to the indicated treatment. **E** WB analysis of the efficacy of the TXNIP knockout. **F** Flow cytometry assay of MMP in vemurafenib-resistant cells with or without TXNIP knockout exposed to the indicated treatments. **G** Seahorse assay in vemurafenib-resistant cells with or without TXNIP knockout exposed to the indicated treatments. **H** Immunofluorescence staining of mt-ROS in vemurafenib-resistant cells with or without TXNIP knockout exposed to the indicated treatments.

Data represent the mean ± SD of triplicates. The differences were analyzed using one-way ANOVA. **P* < 0.05, ***P* < 0.01, and ****P* < 0.001. *ns* non-significant, ko-NC knockout negative control, ko-TXNIP knockout of TXNIP.

**
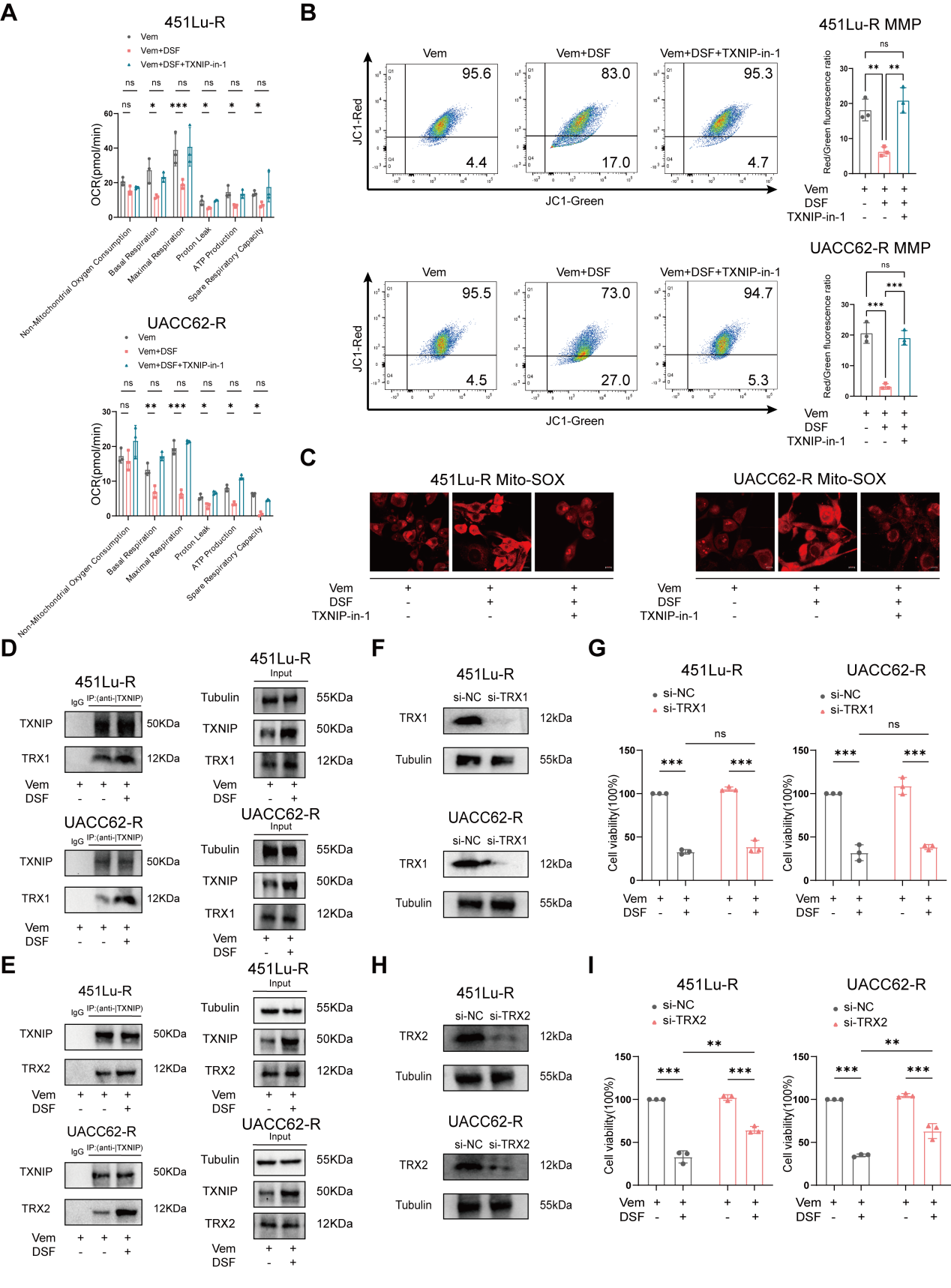
Supplementary Fig. 6 TXNIP provokes mitochondrial oxidative stress in vemurafenib-resistant cells via the interaction with TRX2. A** Seahorse assay of vemurafenib-resistant cells exposed to the indicated treatment. **B** Flow cytometry of MMP levels in vemurafenib-resistant cells exposed to the indicated treatment. **C** Immunofluorescence staining of mt-ROS levels in vemurafenib-resistant cells exposed to the indicated treatment. **D** Co-IP was used to confirm the interaction between TXNIP and TRX1 in vemurafenib-resistant cells exposed to the indicated treatment. **E** Co-IP was used to confirm the interaction between TXNIP and TRX2 in vemurafenib-resistant cells exposed to Vem or Vem+DSF. **F** WB analysis of the efficacy of TRX1 knockdown. **G** CCK-8 assay of vemurafenib-resistant cells with TRX1 knockdown exposed to the indicated treatment. **H** WB analysis of the efficacy of TRX2 knockdown. **I** CCK-8 assay of vemurafenib-resistant cells with TRX2 knockdown exposed to the indicated treatment.

Data represent the mean ± SD of triplicates. The differences were analyzed using one-way ANOVA. **P* < 0.05, ***P* < 0.01, and ****P* < 0.001. *ns* non-significant, si-NC siRNA negative control, si-TRX1 siRNA targeting TRX1, si-TRX2 siRNA targeting TRX2.
