# Supplementary material for: Exploiting mitochondrial dysfunction to overcome BRAF inhibitor resistance in advanced melanoma: the role of disulfiram as a copper ionophore
Source: Cell Death Dis. 2025 Jul 1;16(1):482. doi: 10.1038/s41419-025-07766-y (PMC12216038; doi:10.1038/s41419-025-07766-y)

Fig.5B

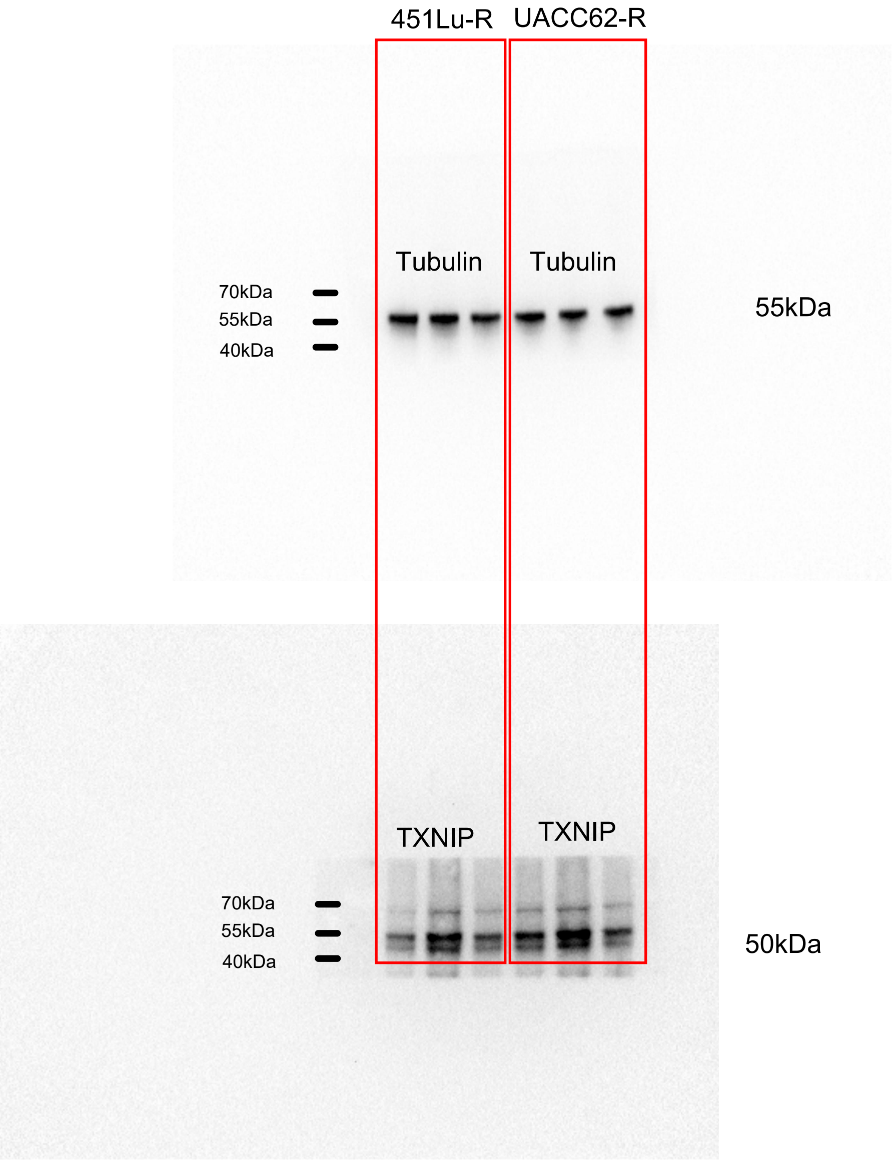
451Lu-R
UACC62-R
Tubulin
Tubulin
70kDa
55kDa
40kDa
55kDa
TXNIP
TXNIP
70kDa
55kDa
40kDa
50kDa

Supplementary Fig.1B

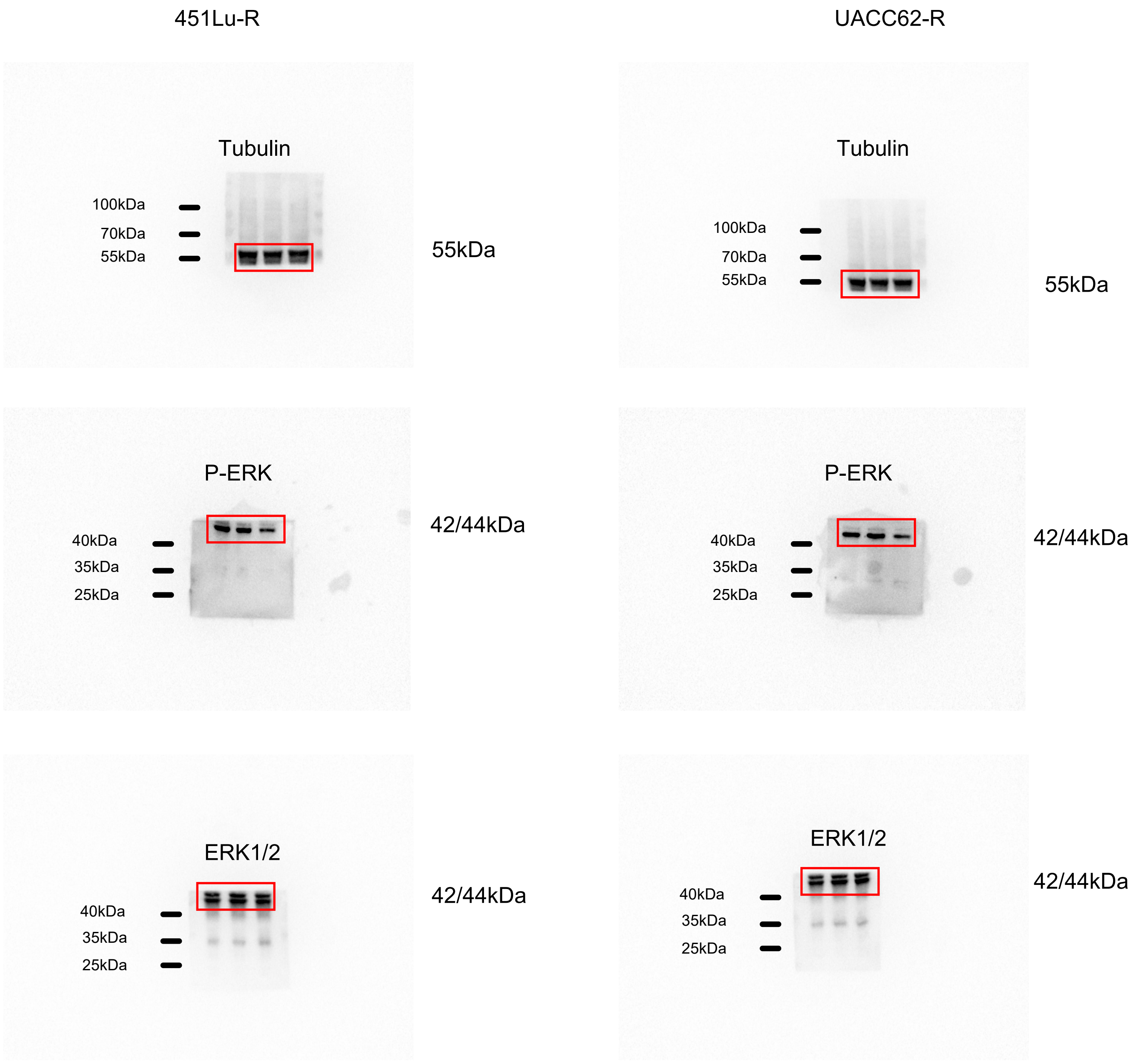

Supplementary Fig.1C

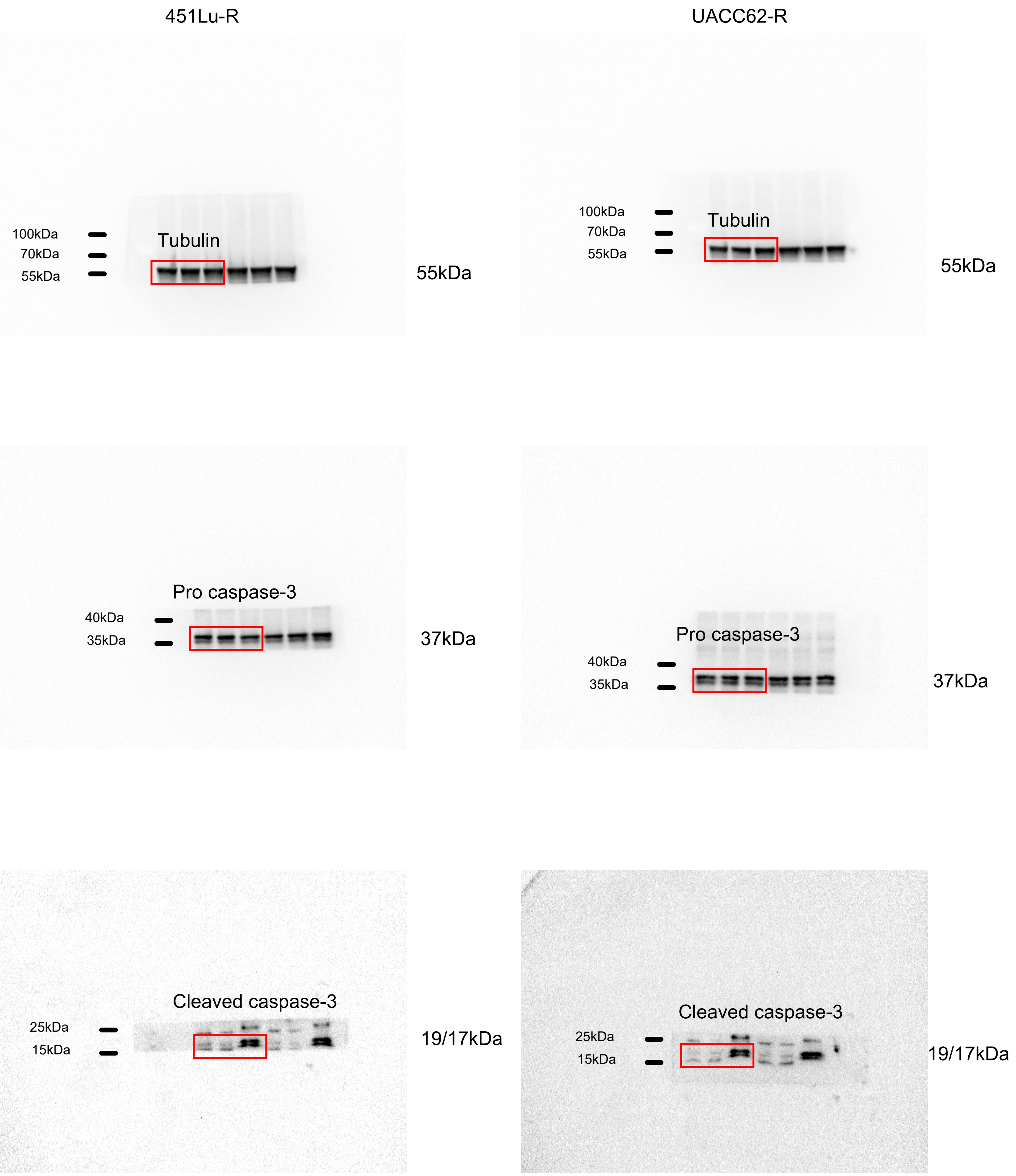

Supplementary Fig.4C

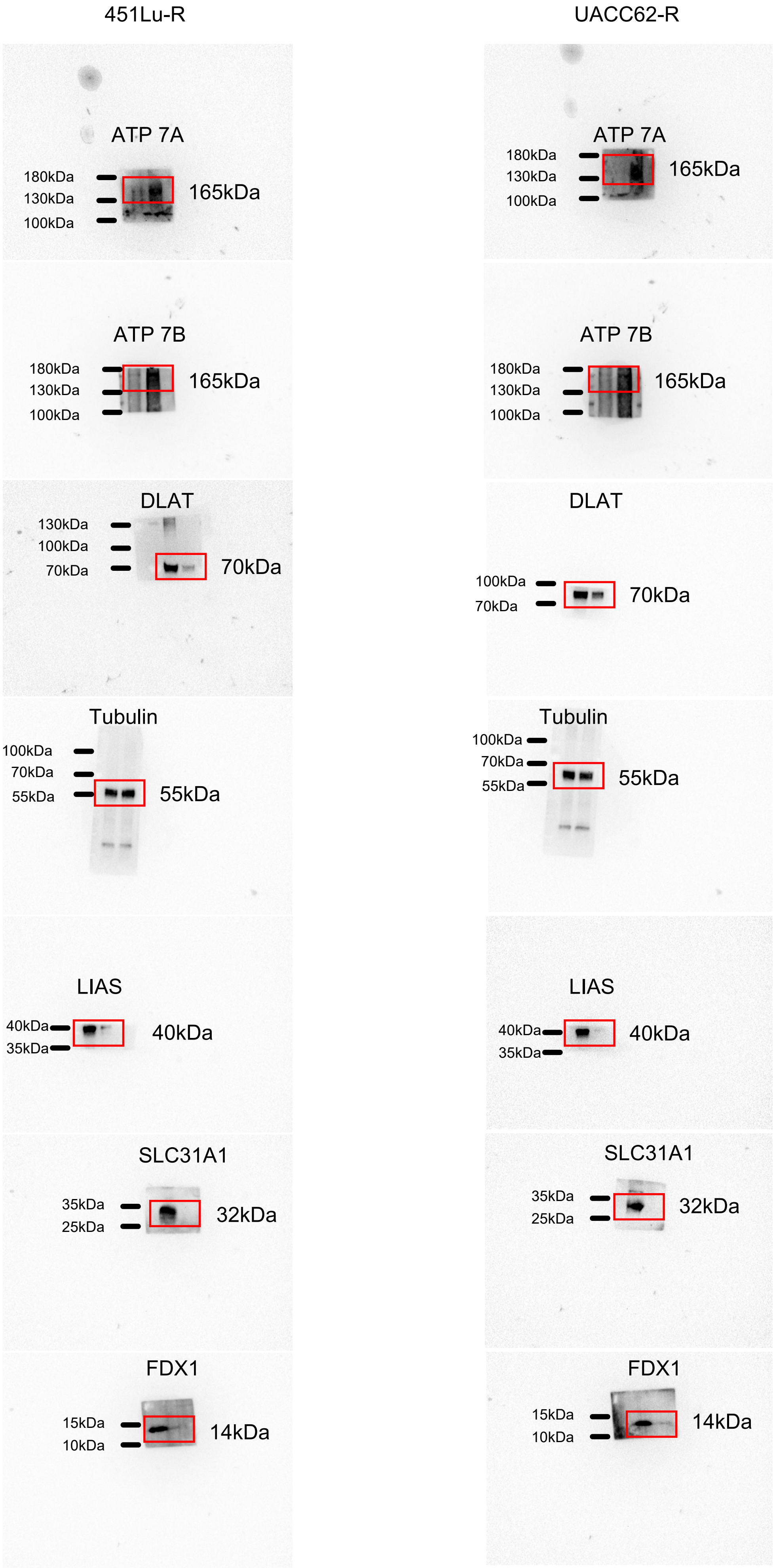

Supplementary Fig.4D

451Lu-R UACC62-R

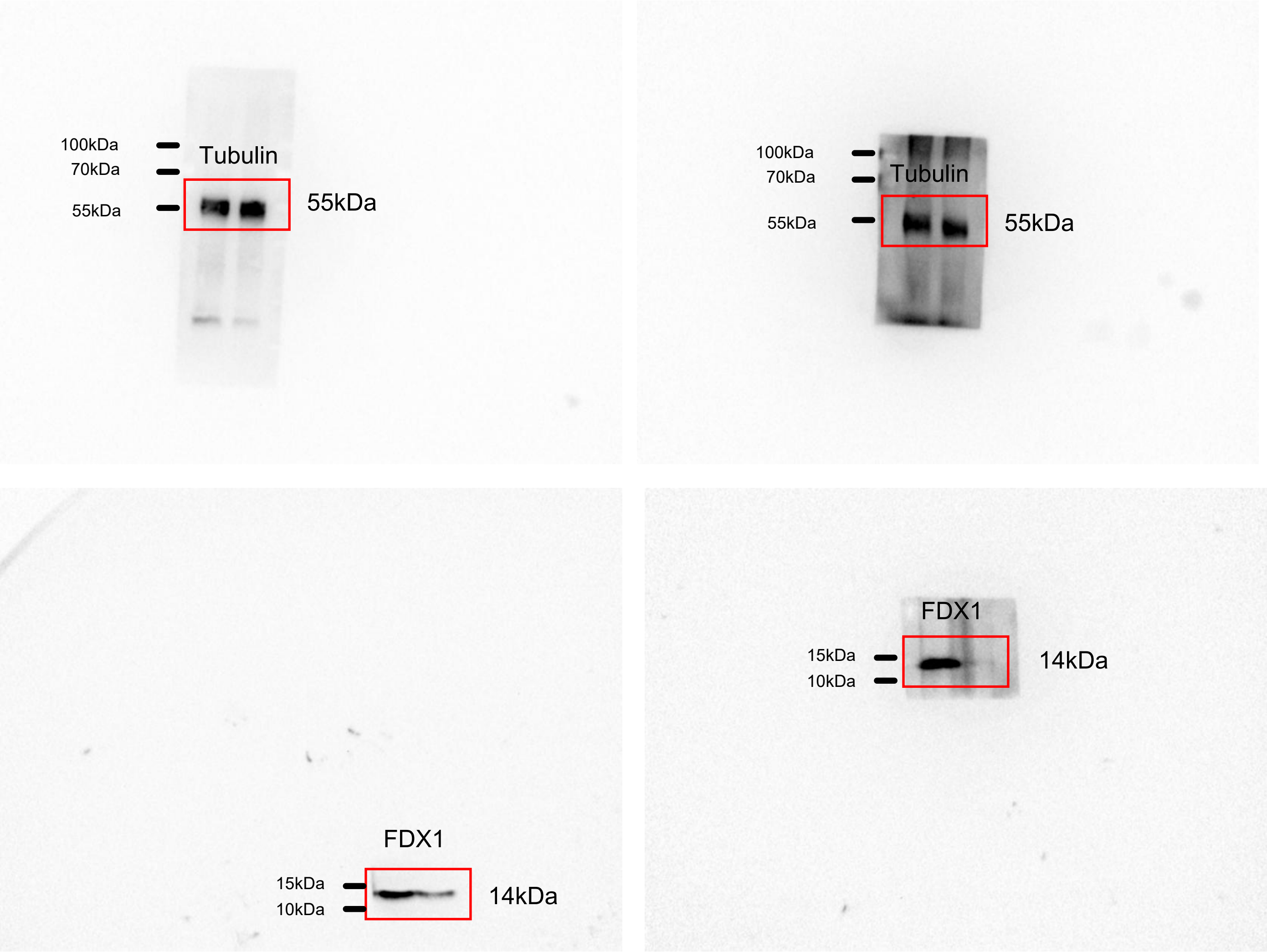

Supplementary Fig.4F

451Lu-R UACC62-R

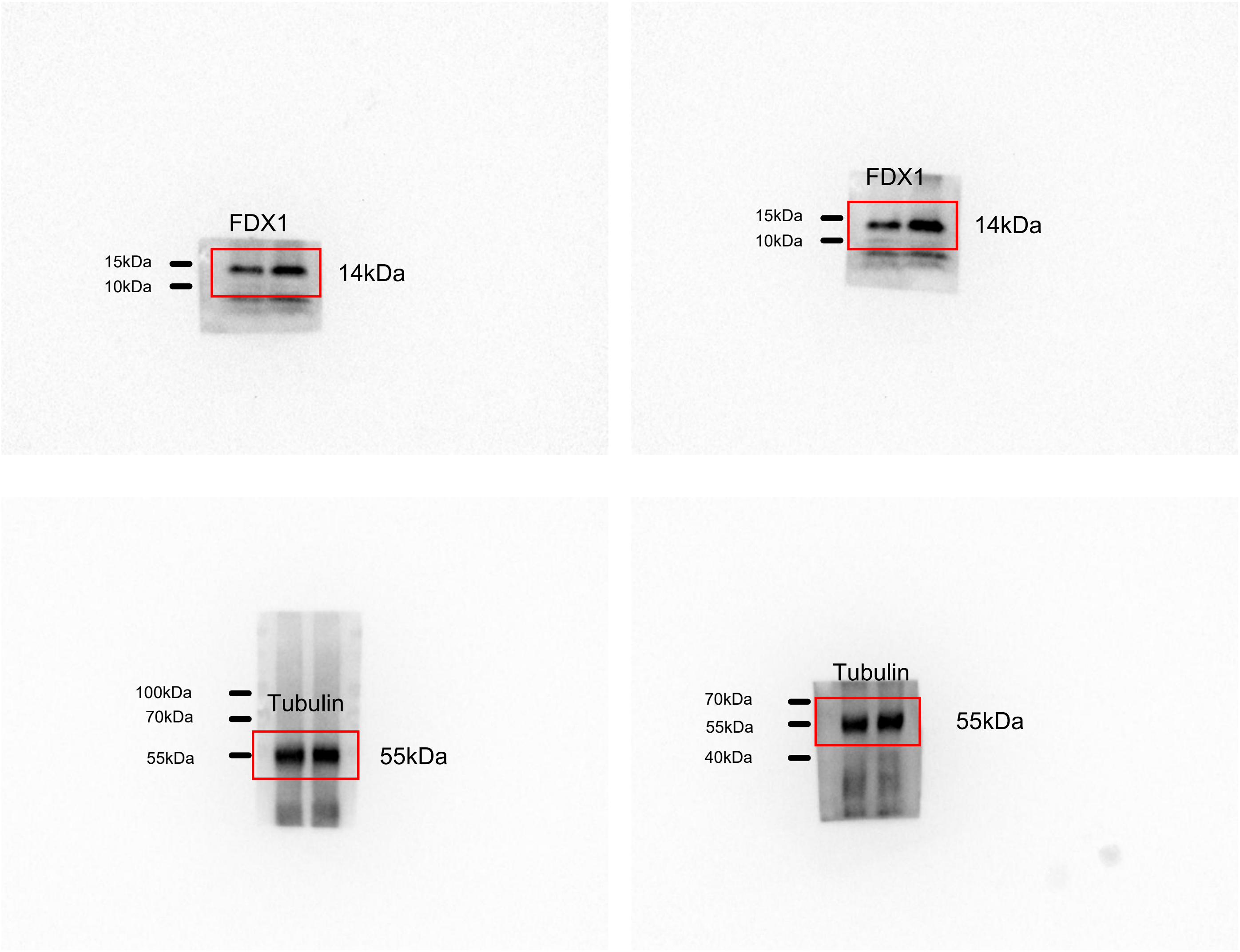

Supplementary Fig.5D

451Lu-R UACC62-R

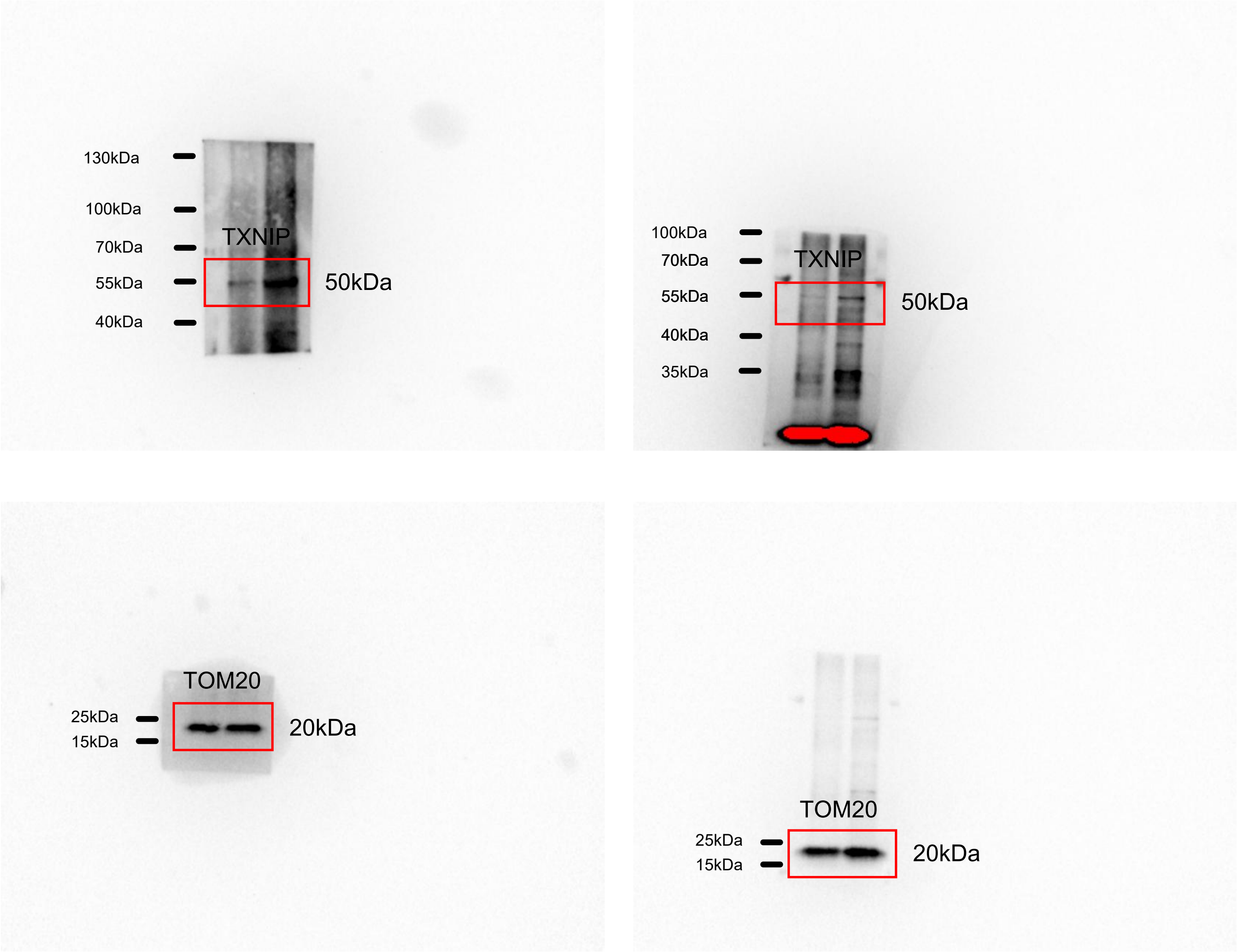

Supplementary Fig.5E

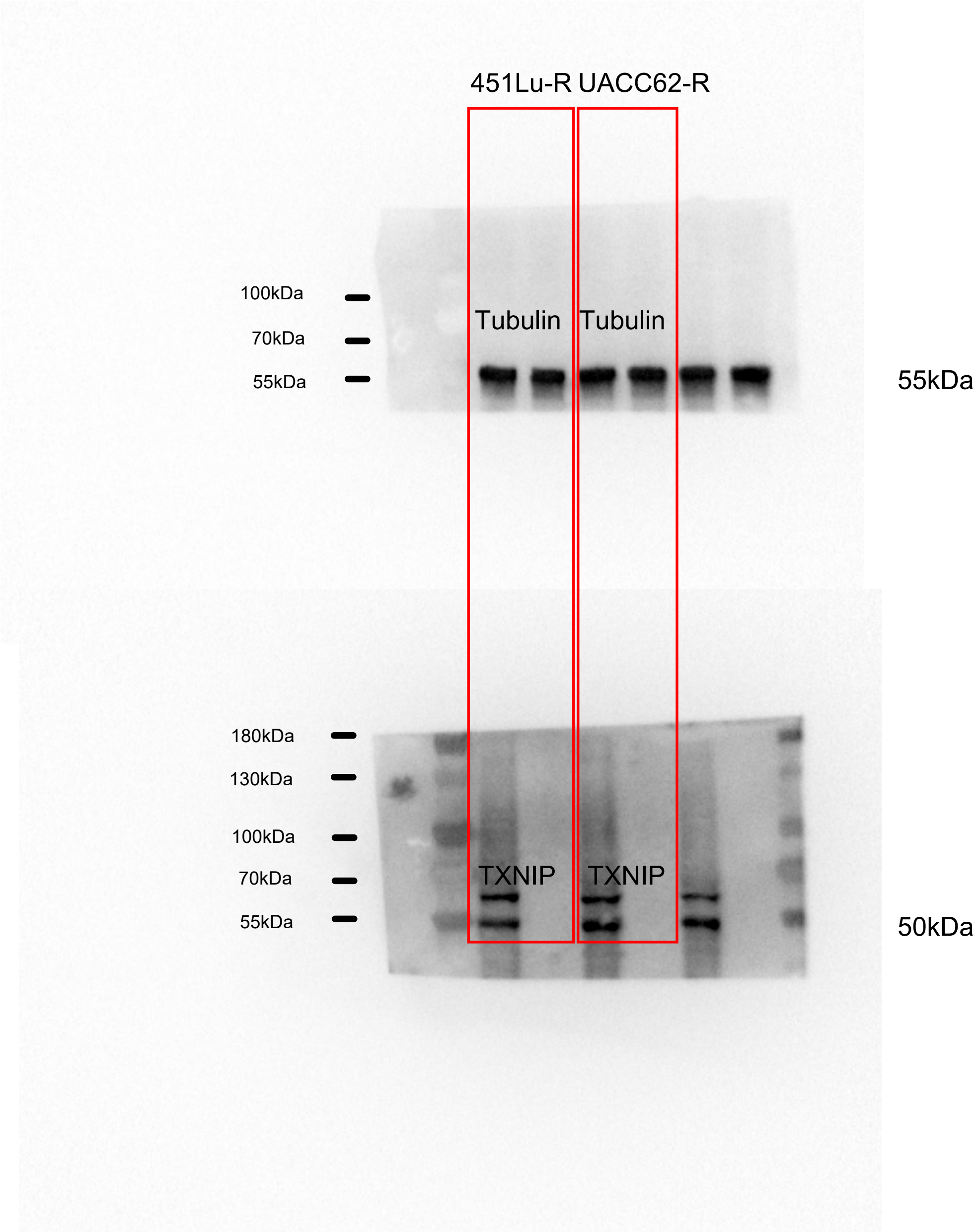

Supplementary Fig.6D

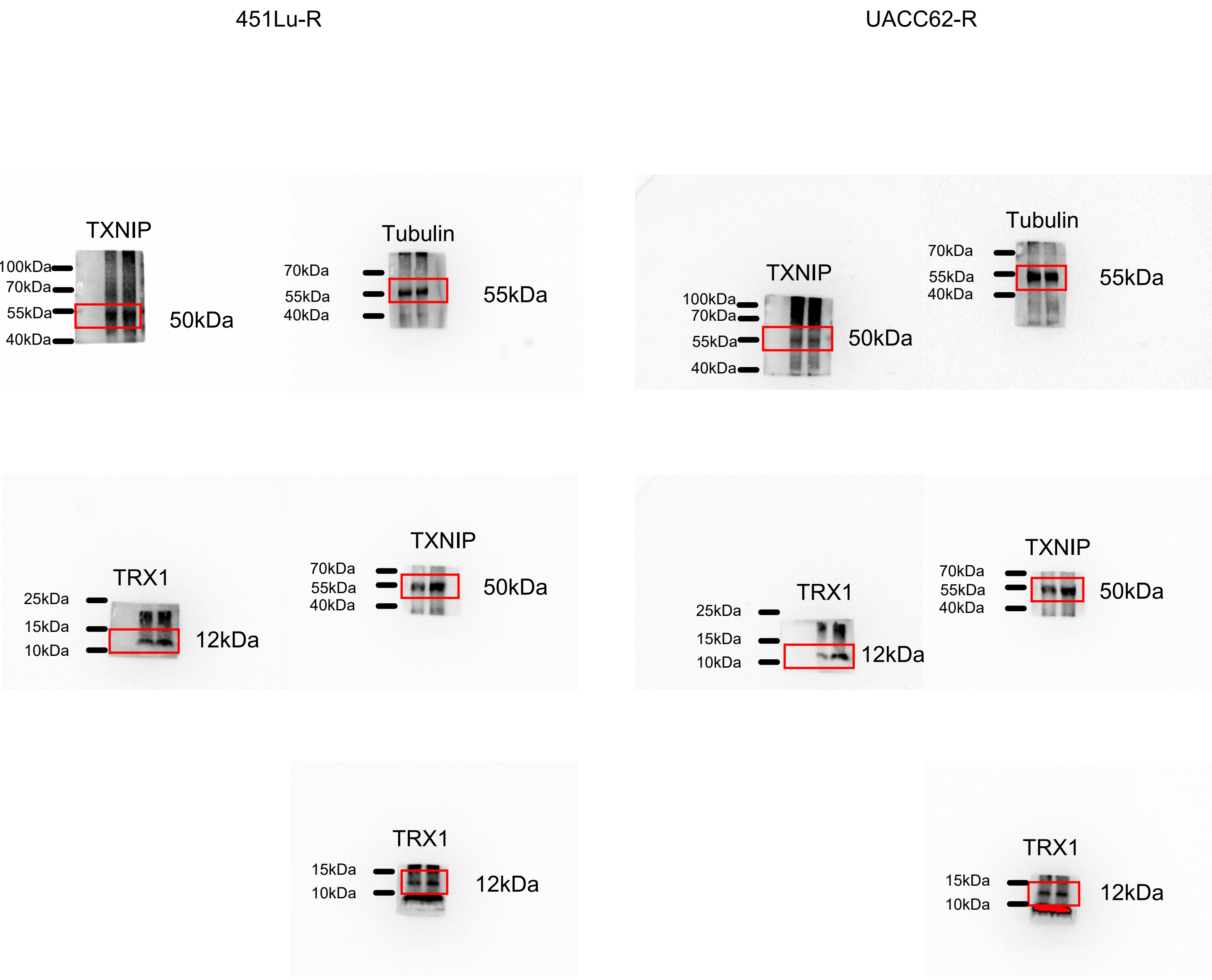

Supplementary Fig.6E

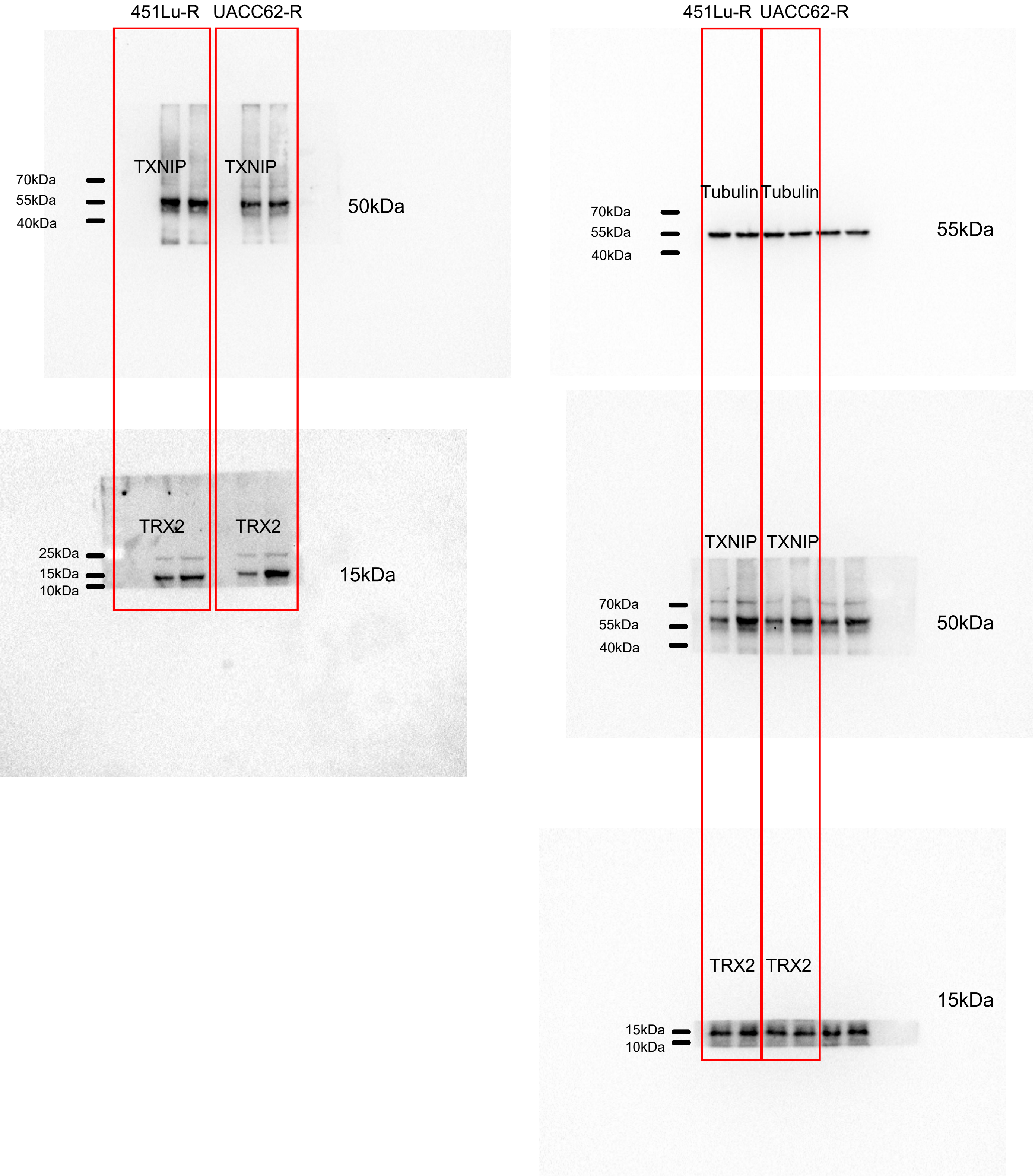

Supplementary Fig.6F

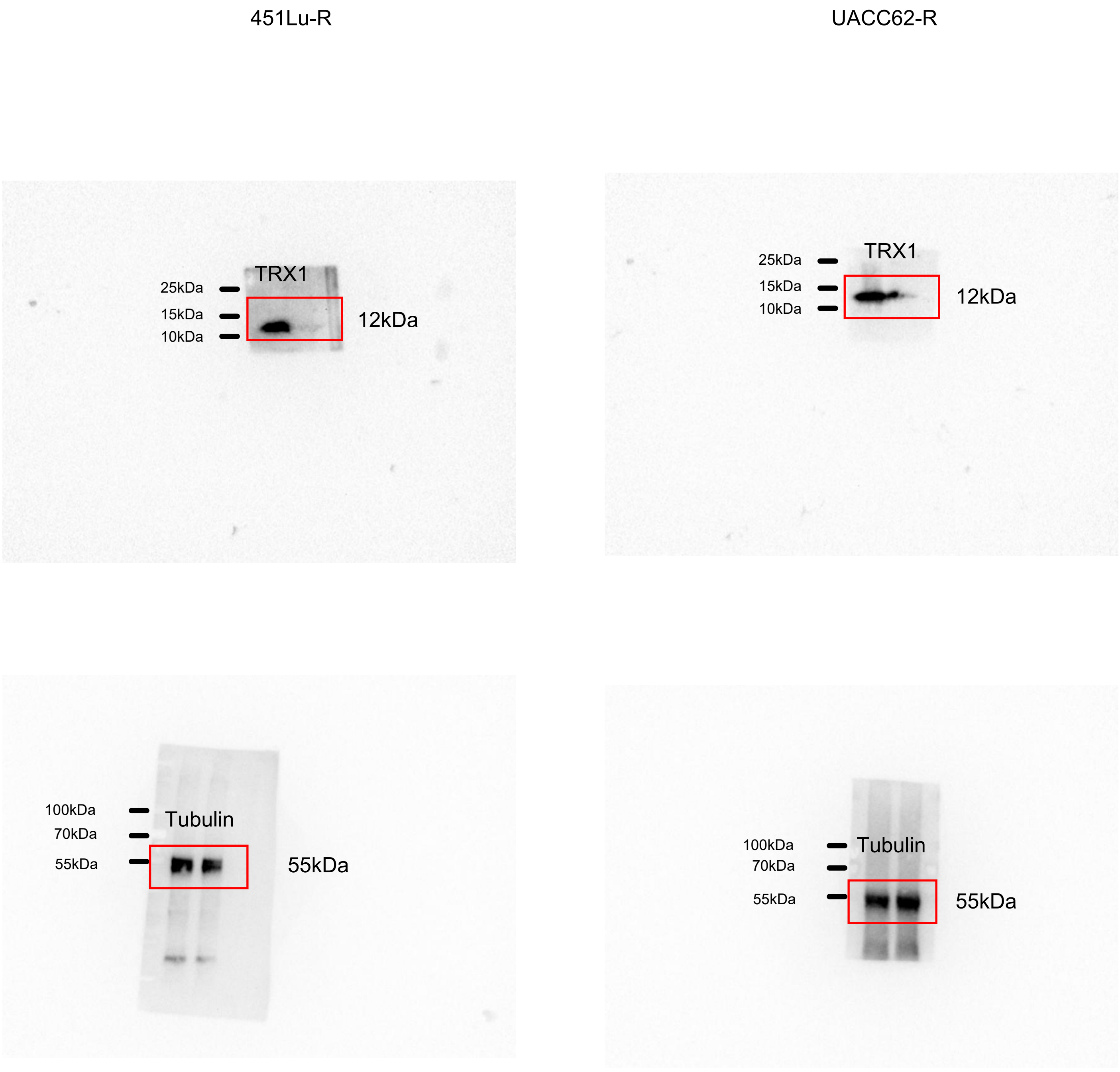

Supplementary Fig.6H

451Lu-R

UACC62-R

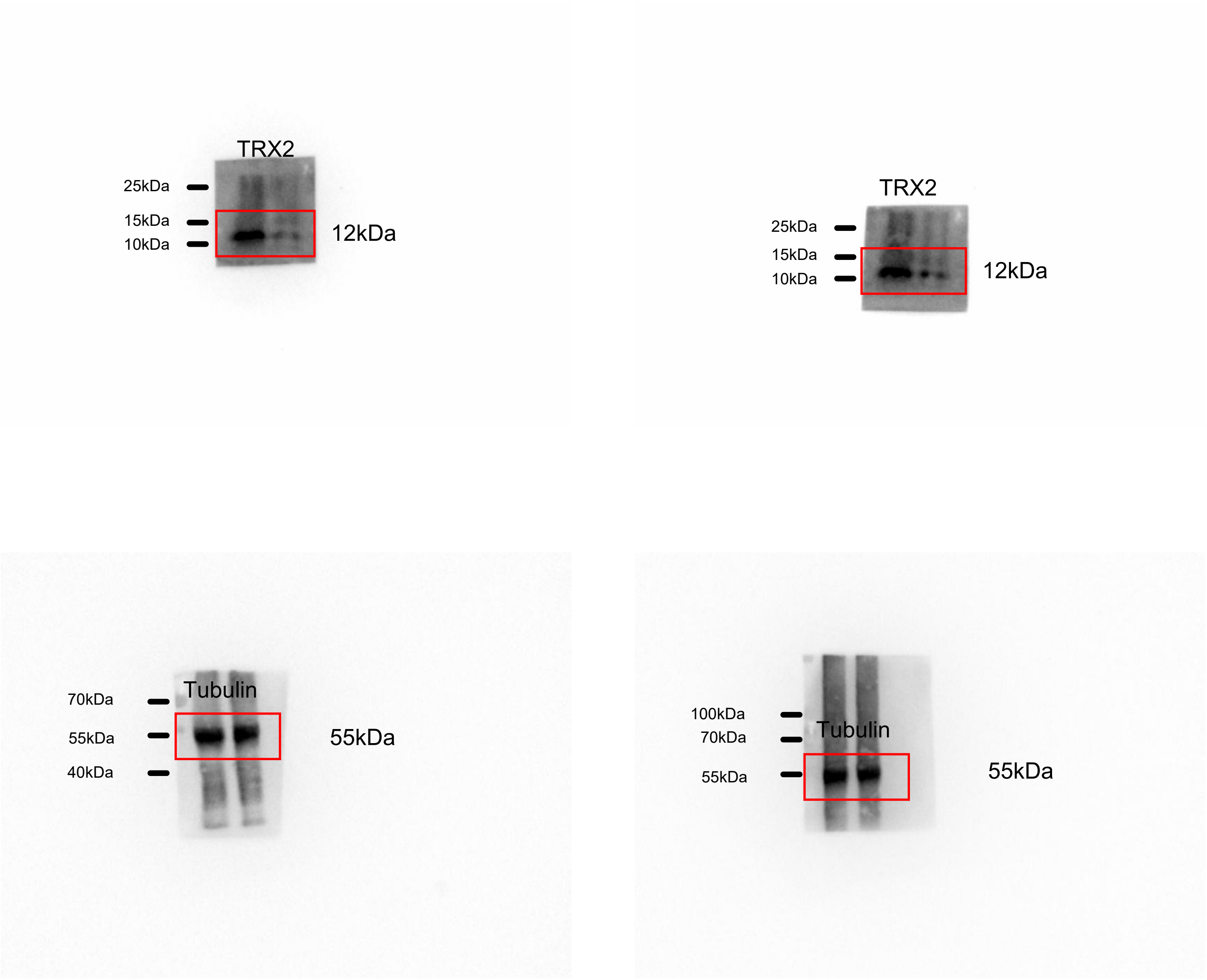

Supplement: Supplementary file 2 — Western blotting [file 41419_2025_7766_MOESM2_ESM.pdf]
